# Piezo1 channel exaggerates ferroptosis of nucleus pulposus cells by mediating mechanical stress-induced iron influx

**DOI:** 10.1038/s41413-024-00317-9

**Published:** 2024-03-29

**Authors:** Ziqian Xiang, Pengfei Zhang, Chunwang Jia, Rongkun Xu, Dingren Cao, Zhaoning Xu, Tingting Lu, Jingwei Liu, Xiaoxiong Wang, Cheng Qiu, Wenyang Fu, Weiwei Li, Lei Cheng, Qiang Yang, Shiqing Feng, Lianlei Wang, Yunpeng Zhao, Xinyu Liu

**Affiliations:** 1 Department of Orthopaedics, Qilu Hospital of Shandong University, Jinan, 250012 China; 2 University of Health and Rehabilitation Sciences, Qingdao, 226000 China; 3 Xiangya School of Medicine, Central South University, Changsha, 410013 China; 4 School of Nursing and Rehabilitation, Shandong University, Jinan, 250012 China; 5 Department of Pediatrics, Cangzhou Central Hospital, Cangzhou, 061011 China; 6 Department of Pediatric Surgery, Qilu Hospital of Shandong University, Jinan, 250012 China; 7 Department of Pathology, Qilu Hospital of Shandong University, Jinan, 250012 China; 8 Department of Spine Surgery, Tianjin Hospital, Tianjin University, Tianjin, 30021 China; 9 The Second Hospital of Shandong University, Cheeloo College of Medicine, Shandong University, Jinan, 250012 China

**Keywords:** Homeostasis, Pathogenesis

## Abstract

To date, several molecules have been found to facilitate iron influx, while the types of iron influx channels remain to be elucidated. Here, Piezo1 channel was identified as a key iron transporter in response to mechanical stress. Piezo1-mediated iron overload disturbed iron metabolism and exaggerated ferroptosis in nucleus pulposus cells (NPCs). Importantly, Piezo1-induced iron influx was independent of the transferrin receptor (TFRC), a well-recognized iron gatekeeper. Furthermore, pharmacological inactivation of Piezo1 profoundly reduced iron accumulation, alleviated mitochondrial ROS, and suppressed ferroptotic alterations in stimulation of mechanical stress. Moreover, conditional knockout of Piezo1 (*Col2a1-CreERT Piezo1*^*flox/flox*^) attenuated the mechanical injury-induced intervertebral disc degeneration (IVDD). Notably, the protective effect of Piezo1 deficiency in IVDD was dampened in *Piezo1/Gpx4* conditional double knockout (cDKO) mice (*Col2a1-CreERT Piezo1*^*flox/flox*^*/Gpx4*^*flox/flox*^). These findings suggest that Piezo1 is a potential determinant of iron influx, indicating that the Piezo1-iron-ferroptosis axis might shed light on the treatment of mechanical stress-induced diseases.

## Introduction

Iron (Fe) is an essential element for humans. Physiological iron concentrations play multiple roles in basic life activities.^1–3^ Iron metabolism is regulated by several proteins, including transferrin receptor 1 (TFR1) and divalent metal transporter 1 (DMT1) for iron transport and uptake, ferroportin (FPN) for intracellular iron export, and ferritin for iron storage.^4–6^ However, pathological iron accumulation can lead to oxidative cellular damage and ferroptosis, a recently described form of cell death involving iron-dependent damage to membrane lipids.^7^ Cellular iron overload, particularly ferrous iron, can lead to lipid peroxidation of fatty acids.^8^ Moreover, iron overload can cause abnormal mitochondrial oxidative phosphorylation pathway, which generates a large amount of reactive oxygen species (ROS) and adenosine triphosphate (ATP).

Cells in multicellular organisms are permanently exposed to mechanical stress, and the ability to generate and respond to mechanical stress is critical to cell behavior and life activities.^9–11^ Various diseases, such as myopathy, heart and kidney failure, leukocyte adhesion defects, and cancer, might develop as a result of the damage caused by these processes.^12^ Mechanical stress can efficiently regulate cellular activity, including cell proliferation, apoptosis, differentiation, and autophagy.^13,14^ Fluid flow shear stress increases intracellular calcium through ion channels, causing the release of intracellular reserves in osteocytes.^15^ Pressure overload in the heart such as pulmonary hypertension can result in cardiac hypertrophy, cardiac fibrosis, and ultimately heart failure.^16^ Immune cells, such as leukocytes, are subjected to various mechanical forces when they leave the bloodstream and migrate through infected and inflammatory regions.^12^ However, the detailed mechanism underlying mechanotransduction (a mechanism by which cells convert mechanical signals into chemical signals) is still not fully understood.

Calcium ions (Ca^2+^) react to mechanical stimulation through the mechanically-sensitive Piezo1 channel (ion channel).^17,18^ Non-sensory cells express Piezo1, which can detect mechanical stresses such as static pressure, shear stress, and membrane stretch. Piezo1 has been shown to mediate the impact of hydrostatic stress on cell fate determination in mesenchymal stem cells.^19–21^ Piezo1 also participates in the progression of renal fibrosis, as well as profibrotic changes and small artery remodeling in hypertensive patients.^22,23^ Moreover, Piezo1 is a mechanical transduction mediator for Ca^2+^-mediated activation of stiff extracellular matrix (ECM) in nucleus pulposus cells (NPCs).^24^ Nonetheless, further research is required to determine the potential role of rigid ECM in Piezo1 activation.

The intervertebral disc (IVD) is a physiological pressure-bearing organ. In addition to maintaining the physiological function of the nucleus pulposus (NP) tissue in people, appropriate mechanical stress also plays a significant role in the internal microenvironment.^25,26^ Frequent bending and twisting, fatigue loading, and strenuous physical activity are some changes related to body posture and weight carrying that can significantly alter the stress on NP tissues. These changes can also cause disturbances in matrix metabolism and accelerate the process of intervertebral disc degeneration (IVDD).^27,28^ There is a positive correlation between changes in the mechanical properties and abnormal changes in the structure and composition of the IVD.^29^ Besides, Piezo1 is upregulated in degenerated NP samples and Piezo1 activation by mechanical stress can accelerate the senescence of human NPCs and IVDD progression via periostin and self-amplifying loop of NF-kB.^30^

In previous experiments, we found that the removal of Ca^2+^ partially alleviated stress-induced cellular ferroptosis in chondrocytes.^31^ Therefore, further studies should investigate the mechanisms underlying the regulation of the cellular behavior by mechanical stress. Detailed information related to mechanical stress mediation of ferroptosis should also be assessed.

Iron is required for ferroptosis and Piezo1 channel is a mechanosensitive ion channel. To date, there are few molecules that can be used for iron influx.^32^ This study aimed to elucidate the function of the Piezo1 channel in IVD and ferroptosis of NPCs. Results showed that Piezo1 is a critical regulator of iron metabolism by directly facilitating iron influx, modulating iron metabolism-associated biomarkers, and affecting the expression of GPX4 (a predominant component in ferroptosis).

## Results

### Piezo1 channel leads to iron overload under mechanical stress

**Fig. 1:**
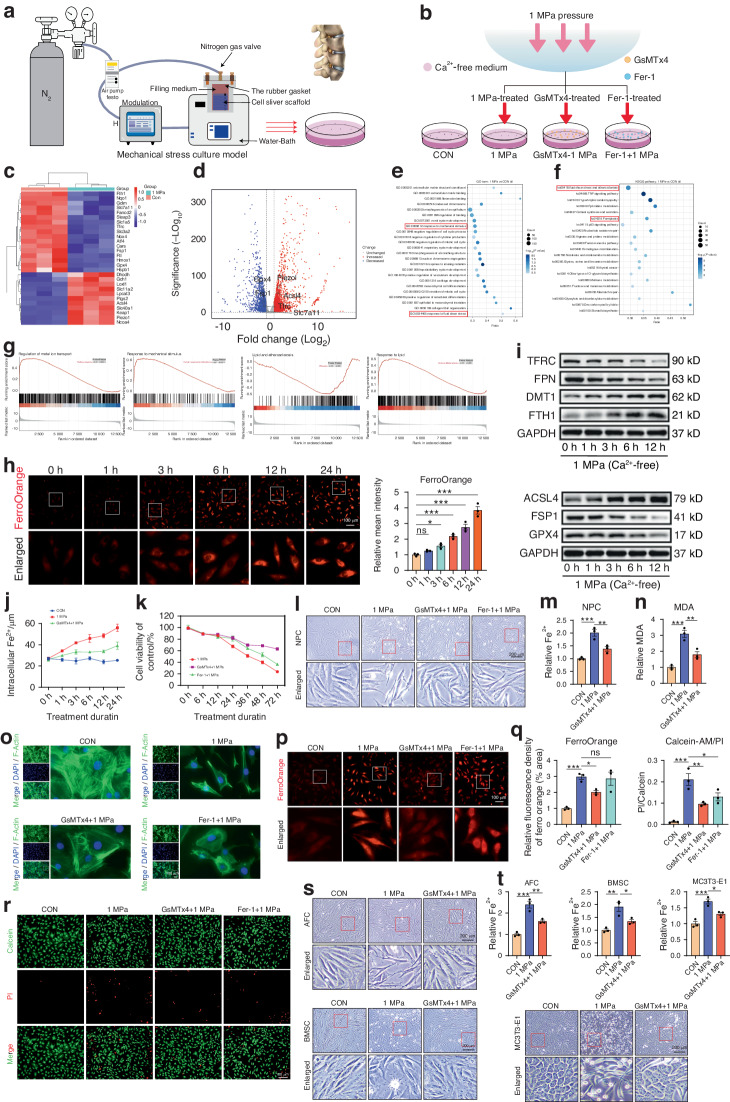
Piezo1 channel leads to iron overload under mechanical stress. **a** Schematic illustration of mechanical stimulation devices. **b** Schematic illustration of the cells treated with different drugs under 1 MPa mechanical stress (*n* = 3). **c** Heatmap illustrating the different genes expression in NPCs. **d** Volcano plot of differentially expressed genes in 1 MPa mechanical stress. **e**, **f** GO a KEGG bubble plots of differentiated pathways in 1 MPa mechanical stress. **g** GSEA analysis showing the changes in regulation of metal ion transport and lipid pathway. **h** Detection of intracellular Fe^2+^ in NPCs at different time-points after stress treatment using FerroOrange and quantitative analysis of relative mean fluorescence intensity (MFI) (*n* = 3). **i** Western blot analysis of iron metabolic markers (TFRC, FPN, DMT1 and FTH1) and ferroptotic markers (ACSL4, FSP1, and GPX4) in different time after 1 MPa mechanical stimulation (*n* = 3). **j** Intracellular Fe^2+^ content are measured by Iron Assay Kit at different time-points (*n* = 3). **k** NPCs were treated with GsMTx4 or Fer-1 under 1 MPa mechanical stimulation for 6, 12, 24, 36, 48, and 72 h (*n* = 3). **l** The morphology of rat NPCs treated with GsMTx4 or Fer-1 under 1 MPa mechanical stimulation. **m** Observation of cytoskeleton in NPCs using Actin Tracker Kit. **n**, **o** Quantitative analysis of relative intracellular Fe^2+^ and MDA content of NPCs treated with 1 MPa mechanical stress with or without GsMTx4 for 24 h (*n* = 3). **p** Detection of intracellular Fe^2+^ in NPCs treated under different treatments for 24 h using FerroOrange and quantitative analysis of relative MFI (*n* = 3). **q**, **r** Cell death/live analysis showing cell death ratio of NPCs (*n* = 3). **s**, **t** Representative morphological changes in AFCs, BMSCs, and MC3T3-E1 after treatmen twith or without GsMTx4 under 1 MPa mechanical stress (*n* = 3). All data are expressed as the mean ± SEM, *n* = 3 replicates from one representative of 3 independent experiments. ns (no significance), *P < 0.05, **P < 0.01, ***P < 0.001

To investigate mechanical stress-induced alterations in NPCs, primary rat NPCs were isolated and cultured with or without 1 MPa mechanical stress in a Ca^2+^-free medium (Fig. 1a, b). Microarray results showed that the Piezo1 was upregulated under mechanical stress (Fig. 1c). Moreover, Piezo1 expression was positively associated with the expression of iron metabolic genes, including ACSL4 and DMT1, and negatively associated with GPX4 and FSP1 (Fig. 1d). GO and KEGG enrichment analysis showed that mechanical stress affected ferroptosis-related pathways (Fig. 1e, f and Fig. S1a, b). GSEA analysis also showed that mechanical stress changed the regulation of metal ion transport and lipid pathway (Fig. 1g, Fig. S1c, d). Furthermore, mechanical stress (1 MPa) significantly changed cell morphology, intracellular Fe^2+^, and the proteins of iron metabolism and ferroptosis in a time-dependent manner (Fig. 1h, i and Fig. S1e–j). NPCs were exposed to 1 MPa mechanical stress with or without GsMTx4 or Ferrostatin-1 (Fer-1) treatment to further demonstrate the role of mechanical stress in iron metabolism. Microarray results revealed that GsMTx4 alleviated the mechanical stress-induced damage (Fig. S2a–i). Moreover, mechanical stress increased intracellular Fe^2+^ levels and cell mortality rate (Fig. 1j, k). Mechanical stress significantly disturbed the morphology of NPCs and increased malondialdehyde (MDA) levels. However, GsMTx4 abolished the iron influx through blockade of Piezo1, suggesting that Piezo1 participates in iron overload in NPCs. Obviously, GsMTx4 significantly alleviated the stress-induced effect than Fer-1 (Fig. 1l–p). Besides, live/dead assay revealed that 1 MPa stimulation markedly promoted cell death in NPCs. However, GsMTx4 and Fer-1 alleviated the above effects (Fig. 1q–r).

To determine whether iron overload was involved in IVD degeneration, NP samples were collected through Pffirmann level II/III or IV/V (Fig. S3a), The samples were then stained with hematoxylin and eosin (HE) staining and Perl’s blue staining. Results indicated that the level of iron increased in the protruded degenerative NP tissues. (Fig. S3b–d). Immunohistochemical results showed that Piezo1 and ferroptosis-related gene ACSL4 was upregulated in patients with a high grade of degeneration, while GPX4 was downregulated, which was consistent with the results of mechanical stress stimulation (Fig. S3e-g). Besides, NP tissues from a needle puncture IVDD model and normal control rats were collected, and Perl’s blue staining indicated that degenerative NPCs displayed an elevated level of iron (Fig. S3h). The effect of mechanical stress on iron metabolism in other types of cells, such as Annulus fibrosus cells (AFCs), bone marrow stromal cells (BMSCs) and mouse embryo osteoblast precursor cells (MC3T3-E1) were also analyzed. Results showed that mechanical stress caused significant morphological damage and increased iron influx in these cells, consistent with results in NPCs (Fig. 1s, t).

### Mechanical stress affects ferroptosis through Piezo1 activation

**Fig. 2:**
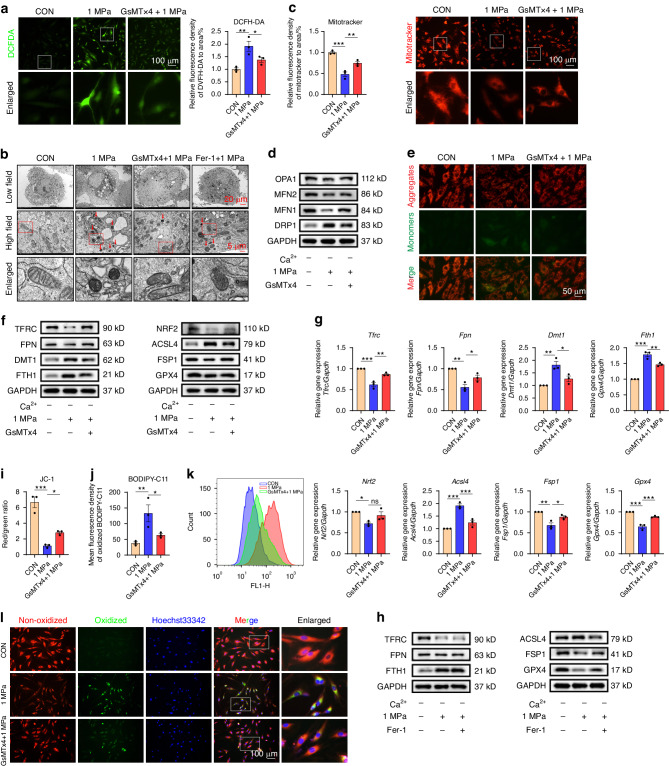
Mechanical stress affects ferroptosis through Piezo1 activation. **a** Detection of intracellular ROS in NPCs treated with GsMTx4 or Fer-1 under 1 MPa mechanical stress for 24 h in a Ca^2+^-free medium. using DCFH-DA and quantitative analysis of relative MFI (*n* = 3). **b** Representative transmission electron microscopy (TEM) images of NPCs treated with different stimulations for 24 h. Arrows indicate shrunken mitochondria. Scale bars, 20 μm (Low field), 5 μm (High field). **c** The mitochondrial function was detected using Mitotracker Kit. **d** WB analysis of mitochondrial function markers OPA1, Mfn2, Mfn1 and DRP1 of NPCs treated with or without GsMTx4 under 1 MPa mechanical stimulation for 24 h and quantification. GAPDH was used as an internal control (*n* = 3). **e** JC-1 assay showing mitochondrial membrane potential of NPCs. JC-1 monomer was stained green, and JC-1 aggregates were stained red. **f** WB analysis of iron metabolic markers and ferroptotic genes of NPCs. **g** PCR analysis of iron metabolic genes *Tfrc*, *Fpn*, *Fth1* and ferroptotic genes *Acsl4*, *Fsp1*, *Gpx4* of NPCs treated with or without GsMTx4 under 1 MPa mechanical stimulation for 24 h and quantification (*n* = 3). **h** WB analysis of iron metabolic markers and ferroptotic markers of NPCs treated with or without Fer-1 under 1 MPa stress for 24 h (*n* = 3). **i, j** Quantitative analysis of relative MFI (*n* = 3). **k** Representative histogram plot for fluorescence of oxidized BODIPY-C11. **l** Lipid ROS in NPCs treated with or without GsMTx4 under 1 MPa mechanical stress for 24 h by using BODIPY-C11. All data are expressed as the mean ± SEM, *n* = 3 replicates from one representative of 3 independent experiments. ns (no significance), *P < 0.05, **P < 0.01, ***P < 0.001

Oxidative stress is associated with pathological processes through several mechanisms. Previous reports have discovered that iron overload leads to oxidative stress and ROS. In this study, 2′,7′-dichlorofluorescein diacetate (DCFDA) assay was performed to measure the amount of ROS. The results revealed that mechanical stress elevated ROS production, while GsMTx4 treatment diminished it. (Fig. 2a). Transmission electron microscope (TEM) showed that mechanical stress affected mitochondrial structure, which exhibited mitochondrial shrinkage and disorganization of mitochondrial crests. However, Piezo1 inhibition alleviated the effect of 1 MPa stimulation (Fig. 2b). Moreover, MitoTracker and JC-1 assays were used to assess mitochondrial function. The expression of mitochondrial function biomarkers, including DRP1, MFN1, MFN2, and OPA1, were also evaluated. As a result, mechanical stress disturbed, while Piezo1 inhibition retained mitochondrial function in a Ca^2+^-free environment (Fig. 2c–e, i). It is reported that Piezo1 facilitates ferroptosis in several conditions.^33^ Therefore, the role of Piezo1 activation in ferroptosis alterations of NPCs was evaluated. Real-time PCR (RT-PCR) and Western blot analyses showed that 1 MPa stimulation altered the expression of iron metabolism markers (TFRC, DMT1, FPN, FTH1, and Hepcidin) and ferroptosis markers (NRF2, GPX4, FSP1, and ACSL4). However, GsMTx4 attenuated the effect of mechanical stress on iron metabolism-related genes, ferroptosis genes, and proteins (Fig. 2f, g and Fig. S4a–c), while a ferroptosis-specific inhibitor Fer-1 only restored the expression of ferroptosis markers. These results indicate that Fer-1 cannot effectively prevent mechanical stress-induced changes in iron metabolism (Fig. 2h, Fig. S4d). Furthermore, the expression of transferrin receptor (TFRC) — a positive ferroptosis marker — was decreased under 1 MPa mechanical stress. Moreover, a lipid peroxidation assay through flow cytometry analysis suggested that lipid ROS levels of NPCs was significantly increased under 1 MPa and was inhibited by GsMTx4, consistent with fluorescence microscope results. (Fig. 2j–l).These findings were in line with the TEM findings.

### Pharmacological activation of the Piezo1 channel significantly increases iron overload

**Fig. 3:**
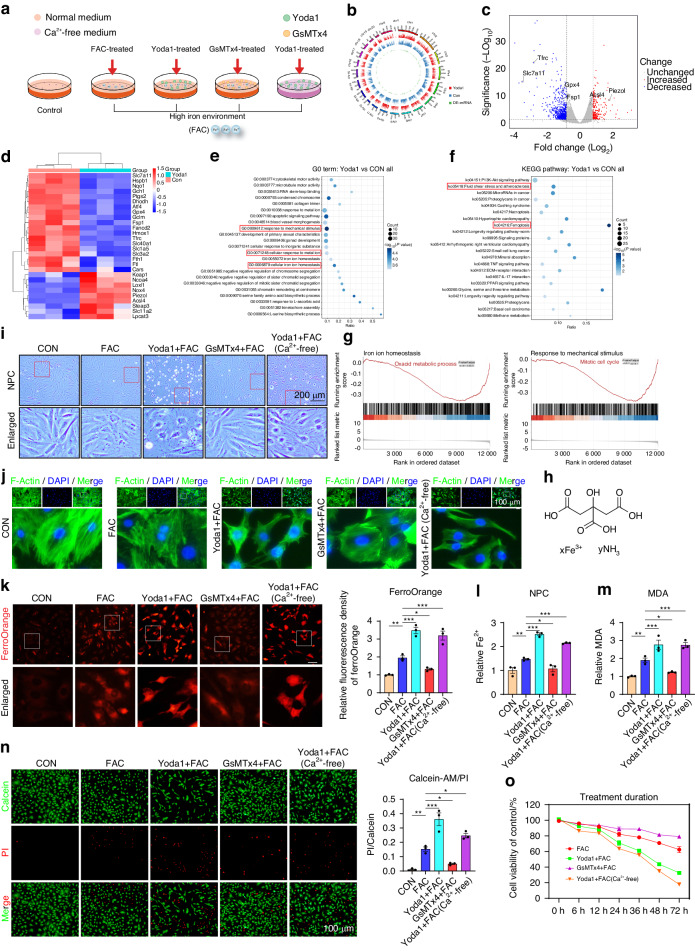
Pharmacological activation of the Piezo1 channel significantly increases iron overload. **a** Schematic illustrations of cells treated with Piezo1 agonist Yoda1 or inhibitor GsMTx4 under high iron environment. RNA sequencing analysis in rat NPCs treated with 10 μmol/L Yoda1 for 24 h in a Ca^2+^-free medium (*n* = 3). **b** Circle map analysis of NPCs. **c** Volcano plot of differentially expressed genes of NPCs treated with yoda1. **d** Microarray heatmap illustrating the different genes expression in NPCs. **e**, **f** GO and KEGG bubble plots analysis in NPCs. **g** GSEA analysis showing the changes in regulation of response to mechanical stimulus and iron ion homeostasis pathway. **h** Chemical structure of FAC. **i** Representative morphological changes of NPCs treated with Yoda1 or GsMTx4 in 100 μmol/L FAC for 24 h. **j** Actin Tracker Kit showing the changes of cytoskeleton in NP cells. **k** Detection of intracellular Fe^2+^ using FerroOrange and quantitative analysis of relative MFI (*n* = 3). **l**, **m** Quantitative analysis of relative intracellular Fe^2+^ and MDA content of NP cells treated with or without Yoda1 or GsMTx4 for 24 h in high iron environment (*n* = 3). **n** The cell death ratio was tested and quantification by cell death/live analysis (*n* = 3). **o** NPCs were treated with different drugs for 6, 12, 24, 36, 48, and 72 h, cell viability was assayed by CCK8 Kit (*n* = 3). All data are expressed as the mean ± SEM, *n* = 3 replicates from one representative of 3 independent experiments. ns (no significance), *P < 0.05, **P < 0.01, ***P < 0.001

To investigate mechanical stress-induced alterations in NPCs further, cells were cultured with Yoda1 (the agonist of Piezo1) in a Ca^2+^-free medium (Fig. 3a). It is well known that Piezo1 activation induces calcium influx, which consequently induces various changes in the cells. In this study, microarray results indicated that Yoda1 altered the levels of genes associated with iron metabolism and ferroptosis (Fig. 3b–d). GO, KEEG and GESA analysis also showed that Piezo1 activation affected iron homeostasis, indicating that the changes in iron metabolism and ferroptosis in NPCs caused by drugs or mechanical stress are similar (Fig. 3e–g and Fig. S5a, b). As a result, we assessed whether Piezo1 directly or indirectly affects iron influx. An extracellular iron overload environment was established using ferric ammonium citrate (FAC) to analyze the association between the Piezo1-mediated metabolism in NPCs and extracellular iron overload. Then rat NPCs were treated with Yoda1 or GsMTx4, and the Yoda1 + Ca^2+^-free medium group was used to eliminate the influence of Ca^2+^ (Fig. 3h, i). F-actin staining results showed that chemical activation of Piezo1 significantly destroyed the cytoskeleton and the intercellular connection. (Fig. 3j). Moreover, FAC increased intracellular Fe^2+^ and MDA levels, especially in the presence of Yoda1. However, GsMTx4 prevented the massive influx of Fe^2+^. This trend was somehow independent of Ca^2+^, as the accumulation of iron was also detected in the Ca^2+^-free medium (Fig. 3k–m). Light microscopy showing the morphology of NPCs found similar results. Additionally, the live/dead assay revealed that Yoda1 markedly promoted cell death in NPCs, which was significantly alleviated by GsMTx4 in a high iron environment independent of Ca^2+^ (Fig. 3n). CCK-8 results also showed that iron environment damaged the cell viability, while Yoda1 greatly accelerated cell death in a time-dependent manner (Fig. 3o).

### Piezo1 channel-mediated iron overload leads to mitochondrial dysfunction, oxidative stress, and lipid peroxidation

**Fig. 4:**
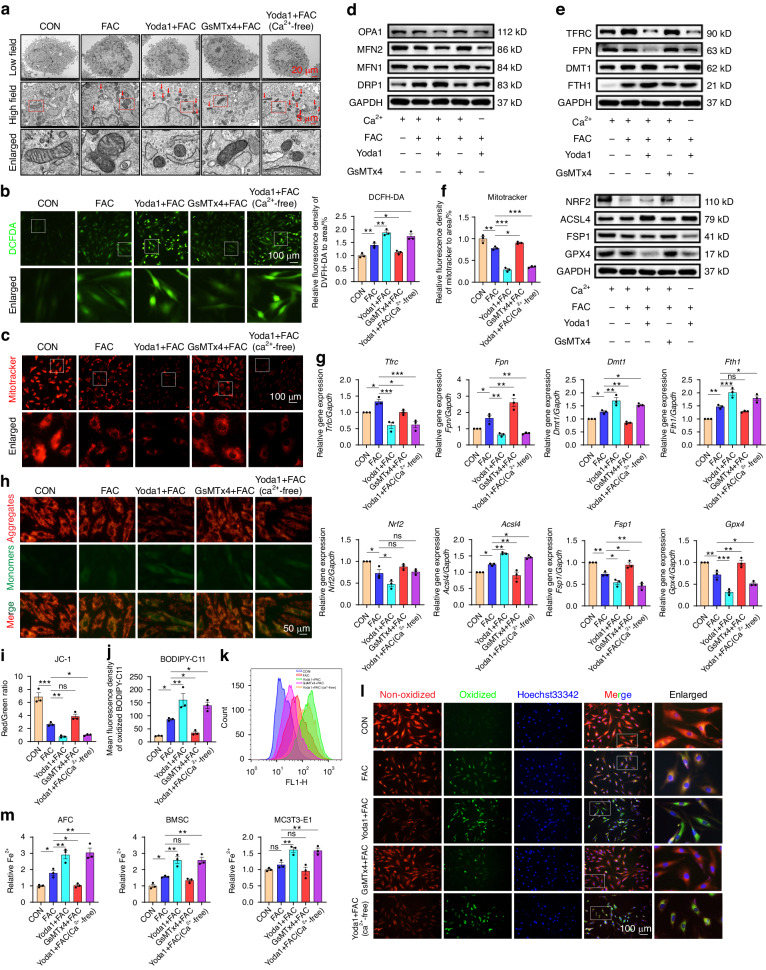
Piezo1 channel-mediated iron overload leads to mitochondrial dysfunction, oxidative stress, and lipid peroxidation. **a** Representative TEM images of of NPCs exposed to the high iron environment, treated with Yoda1 or GsMTx4 for 24 h. Arrows indicate shrunken mitochondria. **b** Intracellular ROS detection by DCFH-DA and quantitative analysis of relative MFI. **c** Mitochondrial function of NPCs evaluated by Mitotracker. **d**, **e** WB analysis of mitochondrial functional markers, iron metabolic markers and ferroptotic markers of NPCs. (*n* = 3). **f** Quantitative analysis of relative MFI of Mitotracker. **g** PCR analysis of iron metabolic genes and ferroptotic genes of NPCs treated with different chemical stimulations for 24 h and quantification. (*n* = 3). **h** Mitochondrial membrane potential of NPCs was assessed through JC-1 assay. **i**, **j** The relative MFI was analysed by ImageJ software (*n* = 3). **k** Representative histogram plot for fluorescence of oxidized BODIPY-C11. **l** Intracellular lipid ROS of NPCs evaluated by C11 BODIPY 581/591. **m** Iron Assay Kit show the Intracellular Fe^2+^ content of different types of cells treated with different chemical stimulations for 24 h (*n* = 3). All data are expressed as the mean ± SEM, *n* = 3 replicates from one representative of 3 independent experiments. ns (no significance), *P < 0.05, **P < 0.01, ***P < 0.001

To identify the association between Piezo1-mediated iron influx and subsequent changes in NPCs, rat primary NPCs were exposed to the high iron environment, and treated with Yoda1 or GsMTx4. ROS production, lipid ROS, mitochondrial structure, mitochondrial function, and mitochondrial function biomarkers were also assessed. TEM analysis showed that Piezo1 activation affected mitochondrial structure. However, the inhibitor of Piezo1 alleviated the detrimental effects of iron stimulation (Fig. 4a). DCFDA assay revealed that Yoda1 elevated ROS production while GsMTx4 treatment decreased ROS production (Fig. 4b). Furthermore, Yoda1 caused the disorganized function of NPCs, while Piezo1 inhibition greatly improved these alterations in a high iron environment (Fig. 4c–i, Fig. S5c–g). Similarly, Piezo1 activation changed gene and protein levels of the indicated groups. Notably, TFRC was up-regulated in the high iron environment, while GsMTx4 slightly reduced the expression. However, TFRC was significantly down-regulated in the Yoda1 group, even after removing Ca^2+^ (Fig. 4e). Flow cytometry analysis suggested that lipid ROS levels significantly increased after Piezo1 channel activation, consistent with fluorescence microscope results (Fig. 4j–l). Moreover, Yoda1 caused significant morphological damage and increased intracellular Fe^2+^ levels in AFCs, BMSCs and MC3T3-E1 (Fig. 4m, Fig. S6a–c). Since GsMTx4 is a non-specific agonist of Piezo1, we treated cells with Yoda1 and FAC in the presence of GsMTx4. Furthermore, rat *Piezo1*-siRNA was also used to validate subsequent results. WB and fluorescent staining images showed blocking Piezo1 channels with GsMTx4 or si-*Piezo1* in the presence of Yoda1 and FAC attenuated the effects of Yoda1 (Fig. S7a–k). These results indicate that Piezo1 activation plays an important role in ferroptosis occurrence.

### Piezo1-induced iron influx is independent of TFRC

**Fig. 5:**
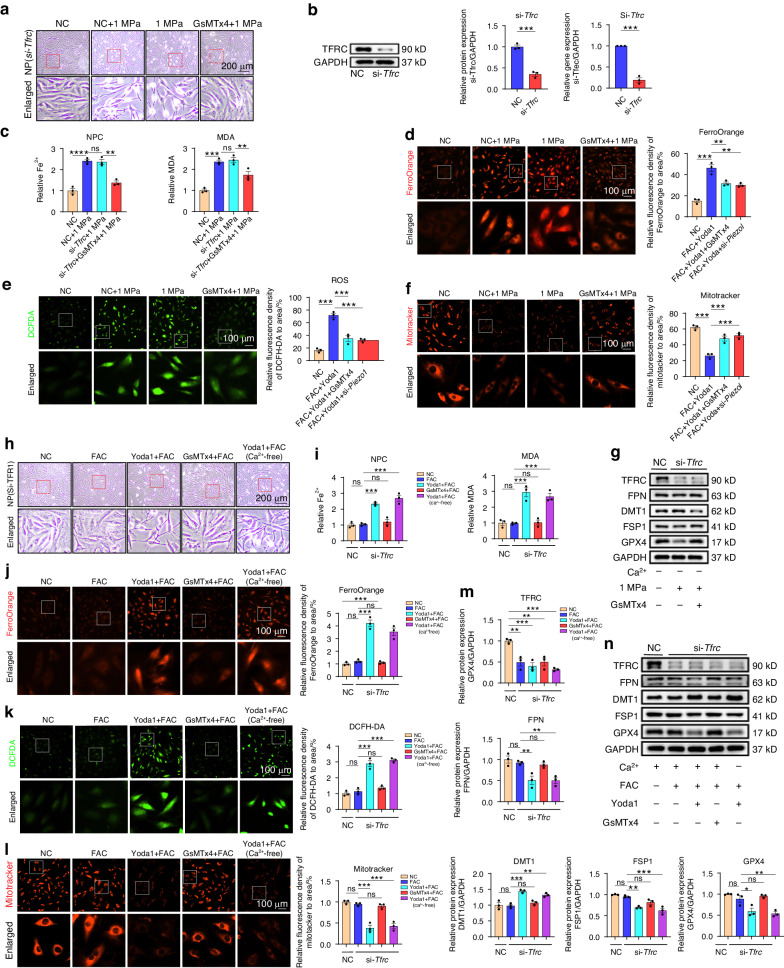
Piezo1-induced iron influx is independent of TFRC. Rat NPCs treated with or without GsMTx4 under 1 MPa mechanical stimulation for 24 h after *Tfrc*-siRNA transfection. **a** The morphology of rat NPCs after transfection. **b** PCR and WB analysis of NPCs and quantitation (*n* = 3). **c** Quantitative analysis of relative intracellular Fe^2+^ and MDA. **d**–**f** Representative images of NPCs were taken using FerroOrange, DCFH-DA and Mitotracker after transfection (*n* = 3). **g** Western blot analysis of TFRC, FPN, DMT1, FSP1 and GPX4 in NPCs treated with mechanical stimulation for 24 h after transfection with si-NC and si-*Tfrc* (*n* = 3). Rat NPCs treated with or without the stimulation of Yoda1 or GsMTx4 under a high iron environment and *Tfrc*-siRNA transfection. **h** Representative cell morphological changes are shown at 24 h of NPCs. **i** Relative intracellular Fe^2+^ and MDA were quantified after transfection (*n* = 3). **j**–**l** Representative images of NPCs were taken using FerroOrange, DCFH-DA and Mitotracker. The relative MFI was analysed by ImageJ software (*n* = 3). **m**, **n** Western blot analysis of NPCs treated with mechanical stimulation for 24 h after transfecting and quantitation (*n* = 3). All data are expressed as the mean ± SEM, *n* = 3 replicates from one representative of 3 independent experiments. ns (no significance), *P < 0.05, **P < 0.01, ***P < 0.001

Microarray, RT-PCR, and Western blot analyses showed that Piezo1 activation by mechanical stress or chemical drugs significantly altered the expression levels of iron metabolism biomarkers, suggesting that Piezo1 may be related to the iron metabolism signaling pathway. TFRC is a well-accepted contributor to the iron influx. In this study, *Tfrc* was knocked down (KD) using *Tfrc*-siRNA transfection to investigate whether Piezo1 can directly facilitate iron influx independent of TFRC. Results showed that the KD efficiency was up to 90% (Fig. 5a, b). Surprisingly, *Tfrc* KD did not protect the normal morphology of the cells treated with mechanical stress. Furthermore, *Tfrc* KD did not relieve stress-induced Fe^2+^ influx, MDA and ROS increase, and mitochondrial dysfunction (Fig. 5c–f). Moreover, the alterations of iron metabolism-related and ferroptosis markers were not mitigated (Fig. 5g, Fig. S8a–c). Interestingly, NPCs became insensitive to FAC stimulation when stimulated with chemical drugs in a high iron environment after transfection, as demonstrated by a decreased intracellular Fe^2+^ and MDA levels (Fig. 5h, i). Besides, *Tfrc* KD reversed the Fe^2+^ influx, ROS increase, mitochondrial membrane potential, and mitochondrial dysfunction stimulated by FAC. However, these phenotypes were not changed after Yoda1 treatment in a high iron environment (Fig. 5j–l). Also, *Tfrc* KD alleviated the damage caused by FAC at the protein level. Nevertheless, Yoda1 and the mechanical stress groups still presented the same alterations of iron metabolism-related and ferroptosis markers even after *Tfrc*-siRNA transfection (Fig. 5m, n). Notably, Yoda1 treatment significantly induced iron overload in a normal or calcium-free medium, suggesting that Fe^2+^ can be transferred into the cytoplasm through the Piezo1 channel.

### Deficiency of the Piezo1 channel attenuates IVDD development through GPX4

**Fig. 6:**
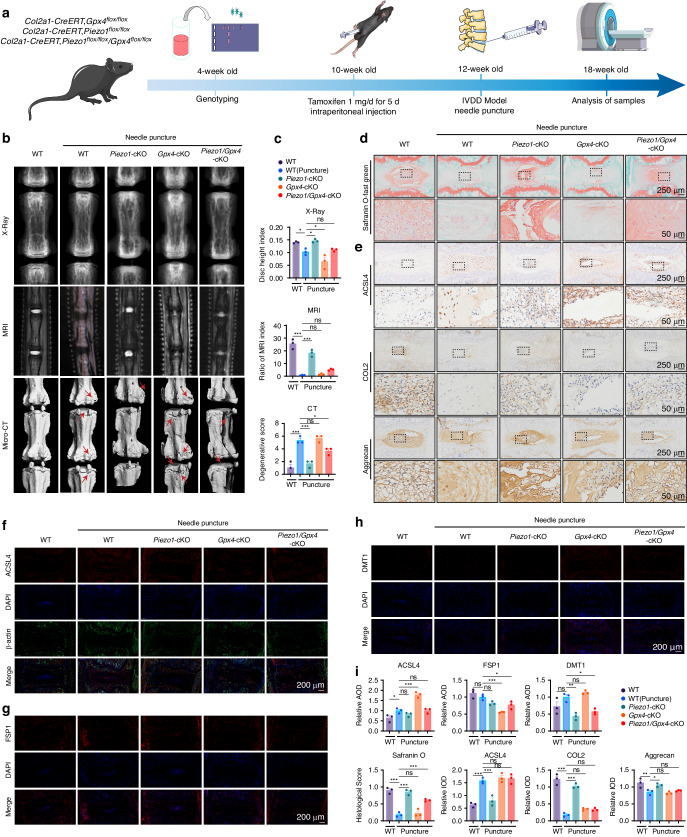
Deficiency of the Piezo1 channel attenuates IVDD development through GPX4. **a** Flowchart of animal experiments on *Piezo1*-cKO、*Gpx4*-cKO、*Piezo1/Gpx4*-cKO mice. **b** The X-Ray, MRI and Micro-CT images for coccygeal disks of mice after needle puncture. **c** Quantitative analysis of X-ray、MRI and Micro-CT (*n* = 3). **d** The Safranine O-Fast Green staining of WT, *Piezo1*-cKO, *Gpx4*-cKO and *Piezo1/Gpx4*-cKO mice. Scale bar, 250 μm. **e** The immunohistochemical assay of ACSL4, COL2 and Aggrecan of mice. Scale bar, 250 μm. **f**–**h** Immunofluorescence staining analysis of ACSL4、DMT1、FSP1 in different mice. Scale bar, 200 μm. **i** Quantitative analysis of the immunofluorescence staining, Safranine O-Fast Green staining and immunohistochemical assay (*n* = 3). All data are expressed as the mean ± SEM, *n* = 3 replicates from one representative of 3 independent experiments. ns (no significance), *P < 0.05, **P < 0.01, ***P < 0.001

**Fig. 7:**
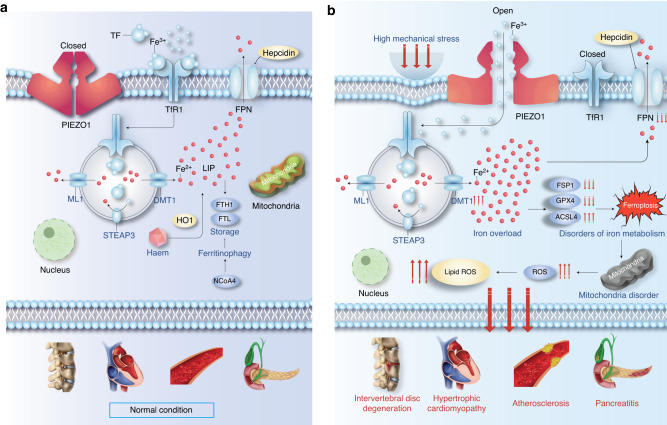
Schematic diagram of iron metabolic pattern. **a** Iron metabolic pattern of cells under normal condition. **b** Iron metabolic pattern of cells under mechanical stress

To evaluate whether the genetic blockade of the Piezo1 channel attenuates the IVDD process in vivo, a needle puncture mouse model was established (Fig. 6a). The present study found that Piezo1 interfered with GPX4 expression and ferroptosis, indicating that Piezo1 may regulate IVDD through GPX4. Therefore, WT, *Gpx4*-cKO (*Col2-CreERT, Gpx4*^*flox/flox*^), *Piezo1*-cKO (*Col2-CreERT, Piezo1*^flox/flox^), and *Gpx4/Piezo1*-cKO mice were used to establish a needle puncture coccygeal IVDD model for further analysis (Fig. S9a–d). MRI for IVD signal, X-ray for IVD height (evaluated by the disc height index; DHI), and micro-CT for the microstructure of vertebral endplate and subchondral (determined via degeneration score) were determined six weeks after needle puncture. Results showed that Piezo1 deficiency attenuated IVDD development, while *Gpx4* deficiency significantly enhanced IVDD development. However, IVDD was more severe in the *Gpx4/Piezo1-*cKO mice than in *Piezo1*-cKO mice. Moreover, DHI assay results were consistent with the MRI findings. Micro-CT suggested that degeneration was severe in *Gpx4*-cKO mice. However, knockout of Piezo1 alleviated IVDD (Fig. 6b, c). Besides, Safranin O staining revealed that Piezo1 deficiency significantly retained the histological integrity and ECM (Fig. 6d). Immunohistochemistry was used to detect the expression of metabolic markers, including COL2, aggrecan, ADAMTS-5, MMP-13, and ferroptosis regulator GPX4. These biomarkers had different expression patterns in IVDD models. Besides, Piezo1 deficiency retained the expression of the metabolic biomarkers. However, the additional loss of *Gpx4* in *Gpx4/Piezo1*-cKO mice abolished the alleviation of IVDD detected in *Piezo1*-cKO mice (Fig. 6e). Moreover, immunofluorescence staining was used to detect the expression of iron metabolic and ferroptosis indicators (ACSL4, DMT1, and FSP1). Analyses showed that the fluorescence intensity of the protective marker FSP1 was enhanced in *Piezo1*-cKO mice, while it was reduced in *Gpx4*-cKO and *Gpx4/Piezo1*-cKO mice. In contrast, the fluorescence intensity of ACSL4 and DMT1 showed the opposite trend (Fig. 6f–i). Additionally, *Gpx4* expression was measured by qPCR in NP cells and NP tissues. The results showed that *Gpx4* was increased in *Piezo1* knockdown cells and in *Piezo1*-CKO mice. But the level of *Piezo1* expression was not statistically different between *Gpx4*-CKO and WT mice (Fig. S9e–g). Taken together, these results demonstrate that Piezo1 can regulate IVDD progression by affecting GPX4 (Fig. 7).

## Discussion

Multiple physiological and pathological processes are closely related to mechanical stress.^14^ Several studies have discovered a cascade of iron overload in different diseases since “ferroptosis” was first introduced in 2012.^34–36^ However, the mechanisms underlying mechanical stress and iron overload are unclear. Herein, the mechanisms by which mechanical stress mediates iron metabolism and ferroptosis were evaluated.

The Piezo1 ion channel was discovered in 2010.^19^ This channel comprises various detachable modules that work together to synchronize the sense-and-conduct of mechanical stimuli using conducting ions. Patapoutian and coworkers showed that constitutive gain-of-function (GOF) Piezo1 allele expression or macrophage expression can result in iron overload.^37^ Although Na^+^, K^+^, Ca^2+^, and Mg^2+^ can pass through the Piezo1 channel, Ca^2+^ has a higher permeability than the other positive ions.^17,38^ In this study, a Ca^2+^-free medium was used to exclude the effect of Ca^2+^. The mechanical stimulation model showed that a nitrogen pressure of 1 MPa could simulate Piezo1 activation, consistent with previous chondrocyte experiments.^31^

The spatial structure of the Piezo1 protein was detected in 2017 using cryoelectron microscopy.^39^ Piezo1 protein has a propeller-like form with three curving “blades” circling the central pore, which is topped by a cap known as the C-terminus. Piezo1’s peripheral blades can be used as a lever-like device when activated.^40,41^ This facilitates chemically and mechanically gated lever transduction pathways, where its iontophoretic and mechanotransduction properties are based. This is a modest conductance, which rapidly inactivates. It is also a low-threshold (1–3 mN/m) protein channel.^42^ Interestingly, the changes in iron accumulation and iron metabolism of protein markers can be observed within the first three hours after treatment with 1 MPa mechanical pressure. This rapid regulation may be related to the specific structure and low-threshold property of Piezo1. However, further studies should investigate whether the short-term massive iron influx process consumes extra energy.

Degenerative diseases of the spine include diseases that involve degeneration of the bony vertebrae and intervertebral disks.^43^ Clinically, most low back pain is caused by degenerative changes in the nucleus pulposus of the intervertebral disc in response to mechanical stress stimulations.^44^ Piezo1 is found in the nucleus pulposus, cartilage endplates and osteoblasts. Physiologically, Piezo1 affects the density and intensity of the vertebral body and disc tissue by influencing cellular differentiation, proliferation or apoptosis, thus playing an important role in spinal growth and development.^45^ Furthermore, Piezo1 can also be used as a second stimulus to directly promote the assembly of NLRP3, activation of caspase-1 and production of IL-1β to mediate the inflammatory response and apoptosis of nucleus pulposus cells.^46^ In this experiment, we used the Piezo1 agonist Yoda1 to simulate mechanical stress, confirming the effect of mechanical stress on the behavior of nucleus pulposus cells via Piezo1, in particular the mechanism of in iron metabolism, providing the molecular basis for the pathological mechanism of spinal degenerative diseases and also new basis, ideas and methods for the prevention and treatment of these degenerative diseases.

Iron-dependent ferroptosis is characterized by reduced glutathione levels and increased lipid peroxidation.^47,48^ Several studies have demonstrated that ferroptosis is involved in neurotoxicity, cancer, renal injury, and iron metabolism-related disorders.^49–51^ Herein, FAC changed iron metabolism and ferroptosis in NPCs, consistent with previous reports.^52^ Interestingly, Piezo1 activation significantly increased these effects in a high iron environment, thus significantly increasing iron influx and ferroptosis. This could be because extracellular iron alters the initial uptake pathway upon Piezo1 channel activation, or the activation of the Piezo1 channel greatly facilitates the classical TFRC pathway, leading to a massive iron influx.

Besides, only a few studies have evaluated iron outside the transferrin pathway. Although it is believed that voltage-dependent Ca^2+^ channels transport non-transferrin-bound iron (NTBI) to cardiomyocytes, a study raised doubts about the function of calcium channels in the cardiac uptake of NTBI.^53^ Researchers confirmed the crucial function of hepatic ZRT/IRT-like protein 14 (ZIP14) in promoting ferroptosis by transporting NTBI in response to iron overload.^54^ It has been demonstrated that TFR1 silencing can prevent ferroptosis caused by erastin.^55^ In this study, *Tfrc* was knocked down with si-*Tfrc* to exclude the classical pathway. The results showed that intracellular iron overload was reduced in the FAC group, while the mechanical stress or Yoda1 group still presented iron overload phenotypes. Therefore, these results indicate that there is a specific mechanical stress-related iron transport mechanism, independent of TFRC.

Almost all circulating iron is physiologically coupled to TF/TFR1, and TFR1 was recently introduced as a specific ferroptosis marker.^56^ Herein, FAC stimulation upregulated TFR1, consistent with previous reports.^57^ However, TFRC was downregulated in NPCs after Piezo1 activation, even in a high iron environment, suggesting that mechanical stimulation does not directly lead to iron influx via TFR1 upregulation. This could be because of a sudden and massive iron inflow through the Piezo1 channel. As a result, the organism initiates endogenous defense mechanisms against iron overload. Nevertheless, this effect is extremely limited, leading to a Fenton reaction of large amounts of Fe^2+^ in the cells, resulting in changes in downstream molecules and eventually ferroptosis. This abnormal phenomenon also supports the idea that Piezo1 is an iron transport channel.

Fer-1 is a first-generation inhibitor of ferroptosis. It has been demonstrated that Fer-1 improves heart function in animal models of acute or chronic illnesses since the anti-ferroptotic impact of Fer-1 was originally noted.^58^ In this study, Fer-1 rescued morphological changes in the mitochondria and restored some downregulated ferroptosis markers, especially typical markers, (GPX4, FSP1, and ACSL4). However, the expression of markers related to iron metabolism levels (TFRC, FPN, and FTH1) was not significantly different after Fer-1 treatment under 1 MPa mechanical stress. Furthermore, the specific Piezo1 inhibitor GsMTx4 alleviated the effect on all the aforementioned markers, lipid peroxidation, and mitochondrial levels under mechanical loading conditions. Possible explanations for this difference could be that ferroptosis occurs under mechanical stress, and the mechanical stress-induced iron influx is a unique mechanism. However, ferroptosis inhibitors, such as liproxstatins, CoQ10, and deferoxamine, have different mechanisms of action, warranting further analysis.^59–61^

Mechanotransduction research entered a new era with the identification and demonstration of Piezo1 channels, which has greatly advanced the understanding of its physiological and pathophysiological significance of this process, human diseases, biophysical properties, and molecular mechanisms.^62,63^ Piezo1 has a physiological role as a mechanotransducer necessary for vascular development, remodeling and controlling blood pressure, cell migration and stem cell fate determination.^64–66^ However, the association between Piezo1-related manifestations and iron overload or ferroptosis is unclear. In this study, the intracellular iron content of other cells that may be susceptible to mechanical stress was also evaluated. Specifically, both AFCs, BMSCs, and MC3T3-E1 exhibited iron overload phenotypes after physical or chemical stimulation of Piezo1 channels. These results broaden the scope of the application of these mechanisms. Cancer cells and many more types of cells exposed to stress should also be validated in the future, which will enrich our understanding of the mechanisms underlying the occurrence and development of diseases. Furthermore, iron overload disorders represent a variety of conditions that lead to systemic iron overload and organ damage. Excessive mechanical stress may be a potential contributing factor to iron overload disorders such as hereditary hemochromatosis, osteoporosis, and cancer. The human body has a dynamic systemic structure, and many important life activities are closely related to stress and iron metabolism. Therefore, it is important to study organ- and cell-specific mechanisms.

In this study, we found that Piezo1 is a key regulator of iron metabolism in disc degeneration. However, there are several limitations that should be noted. Although genetic blockade of Piezo1 attenuates the IVDD process in imaging and immunofluorescence, statistical analysis of some molecules on histological sections showed that double cKO mice have a more severe phenotype than *Gpx4-*cKO mice, suggesting that there are more players involved in this process. We still know very little about the precise mechanisms by which *Piezo1* and *Gpx4* regulate NP cells at the level of genes, as described here, or at the level of proteins or lipids. Further studies are therefore needed to understand the effects of mechanical stress so that they can be exploited in the development of IVDD therapies.

In summary, the present study demonstrated that mechanical stress-induced Piezo1 activation plays a crucial role in the regulation of iron metabolism and ferroptosis. This conclusion is based on three main findings. First, the activation of Piezo1 channel causes intracellular iron overload, ultimately leading to ferroptosis in a stress-responsive manner. Second, the activation of Piezo1 channel directly mediates iron influx in a TFRC-independent manner, which can occur in the early stages, accompanied by a feedback downregulation of TFRC. Third, the deletion of Piezo1 or its inhibitor GsMTx4 can reverse iron overload, and Piezo1-iron-ferroptosis axis may be a novel therapeutic approach for mechanical stress-induced diseases.

## Materials and methods

### Patient tissue samples

Human IVD specimens were collected from 10 patients (5 males and 5 females; age range:15–25 years) with idiopathic scoliosis and 12 patients (7 males and 5 females; age range:21–65 years) with lumbar disc herniation. Informed consent was obtained from each participant and the study protocol was approved by the Medical Ethical Committee of Qilu Hospital of Shandong University (KYll-2021 (ZM) −058).

### Cell culture

The rat nucleus pulposus cells (NPCs), rat annulus fibrosus cells(AFCs) and rat adipose-derived stem cells (ADSCs) were extracted from 4-week old rats and cultured in DMEM/F-12 1:1 (Gibico, USA) supplemented with 10% fetal bovine serum (FBS; Gibico) and 1% penicillin/streptomycin (Gibico). MC3T3-E1 cells were purchased from the Chinese Academy of Sciences and maintained in α-MEM (Gibico) supplemented with 10% FBS.

### Mechanical stress culture model

A mechanical compression device was used as previously described. Briefly, the cells were seeded on 24 mm glass slides and placed on a compressed support in a closed chamber filled with complete medium. The medium in the closed chamber was placed in a cell incubator to maintain cell viability. Static stress (1 MPa) can induce apoptosis and change structural characteristics, matrix composition, and gene expression.^67^ Herein, the cells were exposed to mechanical stress of 1 MPa at a frequency of 1 Hz (induced by pneumatic elements; FESTO, Germany). The influence of Ca^2+^ on cells was eliminated using Ca^2+^-free DMEM/F-12 (Basalmedia, China) medium. The cells were pretreated with 1 μmol/L GsMTx4 (M10039, Abmole, USA) or 10 μmol/L Fer-1 (HY-100579, MCE, USA) for 24 h, then cultured in Ca^2+^-free DMEM/F-12 medium and 1 MPa mechanical stress for 24 h. The cells were grouped into four groups as follows: (i) untreated, (ii) 1 MPa (treated with 1 MPa), and (iii) 1 MPa+GsMTx4 (treated with 1 MPa plus GsMTx4, 1 μmol/L, Piezo1 channel inhibitor) (iv) 1 MPa+Fer-1 (treated with 1 MPa plus 10 μmol/L Fer-1, the inhibitor of ferroptosis). The above treatments were conducted in a Ca^2+^-free medium for 24 h. To examine the time profile after application of 1 MPa mechanical stress, specific indicators were selected at six stages (0, 1, 3, 6, 12, and 24 h).

### Chemical stimulation model

To determine the effect of Piezo1 on iron metabolism and ferroptosis of cells, we first established an extracellular high iron environment with 100 μmol/L FAC (F5879, Sigma, USA). Yoda1 reagent (HY-18723, MCE, USA) and GsMTx4 reagent were introduced to the environment with high iron ion concentration. The cells were then grouped as follows: (i) untreated, (ii) FAC (treated with 100 μmol/L FAC), (iii) FAC+Yoda1 (treated with FAC plus 10 μmol/L+Yoda1, Piezo1 channel agonist), (iv) FAC+GsMTx4 (treated with FAC plus 1 μmol/L GsMTx4, Piezo1 channel inhibitor), (v) FAC+Yoda1 (treated with FAC plus 10 μmol/L Yoda1 in Ca^2+^-free medium). These treatments were conducted in a complete medium for 24 h, except for the fifth group. Since GsMTx4 is a non-specific agonist of Piezo1, we treated cells with Yoda1 and FAC in the presence of GsMTx4. Furthermore, rat *Piezo1*-siRNA was also used to validate subsequent results. The samples were collected from rats or cells subjected to different treatments and examined.

### Measurement of iron content

An Iron Assay Kit (ab83366, Abcam, USA) was used to measure intracellular iron content. Insoluble materials were eliminated through centrifugation at 16 000 x *g* for 10 min, then the cells were homogenized on ice with iron assay solution. The supernatant was collected and incubated with the assay buffer at 37 °C for 30 min. An iron probe was then added to each sample and incubated in the dark at 37 °C for 60 min. The absorbance was recorded at OD 593 nm using a microplate reader (Bio-Rad, USA).

### FerroOrange Fe^2+^ staining

Intracellular Fe^2+^ was detected by using an FerroOrange Kit (F374, Dojindo, Japan). According to the instructions, 10 μmol/L FerroOrange was introduced to the cell cultures and incubated for 60 min at 37 °C with 5% CO2. Images were taken under a fluorescence microscope (Olympus, Japan).

### Perl’s Prussian blue stain

Prussian Blue Iron Stain Kit (G1428, Solarbio, China) was used to evaluate the iron concentration in tissue slices. Human NP sections were deparaffinized and rehydrated. Next, slides containing tissue sections were stained with iron staining solutions following the manufacturer’s instructions. The final staining directly correlates with nonchelated iron in the human NP tissue.

### Transmission electron microscopy

Isolated rat NPCs were fixed for 1 h using Electron Microscope Fixative Solution (G1102, Servicebio, China), and then they were re-fixed for 2 h at room temperature with 1% osmic acid in 0.1 mol/L phosphate buffered saline (pH 7.4). The samples were then embedded in epoxy resin and dehydrated using a series of ethanol concentrations. Finally, a transmission electron microscope (HT7700, Tokyo, Japan) was used to view the sections.

### JC-1 assay

The mitochondrial membrane potential was measured using the JC-1 test kit (C2003S, Beyotime, China) following the manufacturer’s instructions. NPCs were stained with a JC-1 staining solution at 37 °C for 20 min after exposure to different stimulations for 24 h. A fluorescence microscope (Olympus, Japan) was used to capture fluorescent images. The ratio of red to green fluorescence was calculated as an indicator of alterations in the potential of the mitochondrial membrane.

Following the manufacturer’s instructions, the JC-1 test kit (C2003S, Beyotime, China) was used to determine the mitochondrial membrane potential. After being exposed to various stimuli for 24 h, NPCs were stained with a JC-1 staining solution at 37 °C for 20 min. Fluorescent pictures were taken using an Olympus fluorescence microscope (Japan). The ratio of red to green fluorescence was estimated as an indicator of alterations in the mitochondrial membrane’s potential.

### MitoTracker assay

MitoTracker Red CMXRos (C1035, Beyotime, China) was applied in line with the instructions as directed to detecte biologically active mitochondria. As shown, rat NPCs were activated. Cells were treated with MitoTracker Red CMXROS working fluid for 30 min at 37 °C in complete darkness after 24 h. A fluorescence microscope was used to capture fluorescence from the cells.

### Detection of lipid peroxidation level

MDA was measured using a Lipid Peroxidation MDA Assay Kit (S0131M, Beyotime, China). Cells were treated in accordance with the kit’s instructions. The reaction solution was added onto the 96-well plate and its absorbance was measured using a microplate reader (Bi-Rad, USA) at 532 nm. C11-BODIPY 581/591 (GC40165, Glpbio, USA) is used to detect lipid peroxidation in living cells. A fluorescent microscope (Olympus, Japan) was used to capture the images after addition of C11-BODIPY 581/591 (10 mol/L) directly into various groups and incubated for 30 min at 37 °C. For flow cytometry analysis, cells were digested and resuspended in 400 µL of serum-free medium with C11-BODIPY C11 (10 μmol/L). After that, NPCs were incubated for 30 min and the nuclei were stained using Hoechst 33342 (C1022, Beyotime, China) for 5 min. The samples were subsequently detected using a flow cytometer (CytoFLEX LX, Beckman Colter) and data were collected from the FL1 channel.

### Cell transfection

The rat *Tfrc-*siRNA, rat *Piezo1-*siRNA and their negative control (NC) were sourced from GenePharma (Shanghai, China). The Lipofectamine 3000 transfection reagent (Thermo Fisher Scientific, USA) was used for cell transfection following the manufacturer’s instructions. The cells were harvested after 48 h of transfection for further experiments. The sequences are listed in Table S1.

### RT-PCR assay

Total RNA was extracted using the TRIzol Reagent (Invitrogen, USA) and reversed using the Evo M-MLV RT Mix Kit (AG11728, Accurate Biology, China). To accomplish RT-qPCR, a SYBR Green PCR master mix (AG11701, Accurate Biology, China) was used. The relative mRNA expression levels of the target genes were determined using the 2^−ΔΔCT^ method with GAPDH serving as the endogenous reference. Table S2 shows a complete list of all the primers utilized.

### Western blotting

Proteins were extracted by RIPA (P0013B, Beyotime, China) with PMSF (AR1192, Boster, China). BCA Protein Assay Kit (P0011, Beyotime, China) were used to quantify proteins. The standard western blotting was performed with primary antibodies against: NRF2 (1:1 000, 16396-1-AP, Proteintech, USA); ACSL4 (1:1 000, 22401-1-AP, Proteintech); FSP1 (1:1 000, 20886-1-AP, Proteintech); GPX4 (1:1 000, ab125066, Abcam, USA); TFRC (1:3 000, ab269513 Abcam); FPN (1:1 000, 26601-1-AP, Proteintech), DMT1 (1:1 000, YN3198, Immunoway, USA); FTH1 (1:1 000, ab183781, Abcam); MFN1 (1:3 000, ab221661, Abcam); MFN2 (1:3 000, ab205236, Abcam); DRP1 (1:3 000, ab184247, Abcam); OPA1 (1:3 000, ab157457, Abcam); Hepcidin (1:1 000, DF6492, Immunoway, USA); GAPDH (1:3 000, 10494-1-AP, Proteintech). Horseradish peroxidase (HRP)-conjugated secondary antibodies were purchased from Abcam (1:5 000, Abcam).

### Animals

All animal experiments in this study were performed in accordance with the International Guiding Principles for Animal Research and were approved by the Laboratory Animal Center of Shandong University. *Col2a1-CreERT* mice were established by and purchased from Cyagen (USA). *Gpx4*^*flox/+*^ mice were created by Cyagen (USA) through ES genome engineering. *GPX4*^*flox/+*^ mice were mated with *Gpx4*^flox/+^ mice to generate *Gpx4*^*flox/flox*^ mice. *Gpx4*^*flox/flox*^ mice were mated with *Col2a1-CreERT* mice to generate *Col2a1-CreERT, Gpx4*^*flox/+*^ mice. *Col2a1-CreERT Gpx4*^*flox/+*^ mice were mated with *Col2a1-CreERT Gpx4*^*flox/+*^ mice to generate *Col2a1-CreERT Gpx4*^*flox/flox*^ mice. Male mice with the *Col2a1-CreERT* and *Gpx4*^*flox/flox*^ genes were used in experiments. Ten-week-old *Col2a1-CreERT Gpx4*^*flox/flox*^ mice were intraperitoneally injected with tamoxifen (1 mg/d for 5 d) (HY-13757A, MCE, USA) to obtain *Gpx4* conditional knockout (*Gpx4*-CKO) mice. *Piezo1*^*flox/+*^ mice were created by Cyagen (USA) through ES genome engineering. Piezo1 conditional knockout (*Piezo1*-cKO) mice were produced by the same way. *Col2a1-CreERT Gpx44*^*flox/+*^ mice were mated with *Col2a1-CreERT Piezo1*^*flox/+*^ mice to generate *Col2a1-CreERT Piezo1*^*flox/flox*^*/Gpx4*^*flox/flox*^ mice. Male mice with the *Col2a1-CreERT* and *Piezo1*^*flox/flox*^*/Gpx4*^*flox/flox*^ genes were used in experiments. Ten-week-old *Col2a1-CreERT Piezo1*^*flox/flox*^*/Gpx4*^*flox/flox*^ mice were intraperitoneally injected with tamoxifen (1 mg/d*5d) (HY-13757A, MCE, USA) to obtain Piezo1/GPX4 conditional knockout (*Piezo1/Gpx4*-cKO) mice. *Col2a1-CreERT GPX4*^*+/+*^ *Piezo1*^*+/+*^ littermates were assigned to the *wild-type* (WT) group. Three-month-old Sprague-Dawley (SD) rats were purchased from the Animal Center of Shandong University. All of the animals were housed under controlled identical specific pathogen-free (SPF) standard environmental conditions (23 ± 2 °C, 12 h light/dark cycle) with free access to food and allowed to move freely. WT and *Gpx4*-cKO mice were fed with water supplemented with Se-Met (2 mg/L, HY-114245, MCE, USA) and maintained on the diet for 8 weeks around establishment of IVD needle puncture model.

### Genotyping

Tail clippings from 4-week-old mice were taken. According to the manufacturer’s instructions, mouse tail DNA was extracted using a One Step Mouse Genotyping Kit (PD101-01, Vazyme, China). To make agarose gels, agar (1.5 g), 100 mL of 2 × Tris-acetate-EDTA buffer (TAE), and 6 μL of Gel Red were mixed and heated. Agarose gel electrophoresis was used to separate the amplified DNA. Amersham Imager 680 (GE, USA) was used to capture the images. Table S3 lists the primers used for amplification (*Gpx4*^*flox*^, *Piezo1*^*flox*^ and *Col2a1-CreERT*).

### IVDD model establishment

To establish an IVDD model in vivo, caudal needle puncture injuries were performed in 12 weeks old WT mice (*n* = 10), *Piezo1*-cKO mice (*n* = 10), *Gpx4*-cKO mice (*n* = 10) and *Piezo1/Gpx4*-cKO animals (*n* = 5). The surgeries were carried out under general anesthesia (2% isoflurane in oxygen) and sterile conditions. After confirming the location of the IVD with a microscope, needle punctures were made to a depth of 50% of the dorsal-ventral width with 26 G syringe needles. Mice were thoroughly observed to verify that there were no surgical complications and were permitted free movement in their cages with access to water and food.

### Magnetic resonance imaging (MRI)

The mice underwent MRI scanning 6 weeks following the initial puncture to assess structural differences and signal intensity changes in sagittal T2-weighted images of IVDs. 3.0 T MRI scanners (GE Signa HDX, USA) were used for the disc imaging evaluation. Mice were restrained in a supine position with their tails straightened. Spin echo repetition time was 2 275 ms; echo time was 80 ms; number of excitations was 8; field of view was 5 cm; slice thickness was 1.5 mm; and there was no phase wrap. T2 intensities and MRI indices (the area of NP multiplied by the average signal intensity) were calculated using the procedures reported in a prior study.

### Micro-CT

The scanning protocol included an isometric resolution of 15 μm, as well as X-ray energy settings of 70 kV and 200 A. A Quantum GX2 scanner (PerkinElmer, USA) was used to measure the microstructure of the vertebrae. Before histological processing, samples were fixed in paraformaldehyde and micro-CT was performed. Each group’s scanned pictures were analyzed at the same threshold to enable for 3-dimensional structural reconstruction of each sample. The degenerative score was calculated using the procedures outlined in a previous study.^68^

### HE and Safranine O staining

Safranine O staining was performed to detect the changes in proteoglycans with HE staining kit (C0105, Beyotime, China) and Safranine O staining kit (G1371, SolarBio, China) according to the manufacturer’s recommended procedure.

### Immunohistochemistry

The IVD tissues were decalcified, embedded in paraffin, and cut into 5 m slices after being fixed in 4% paraformaldehyde. The paraffin slices were antigen-repaired with citric acid (pH 6.0), blocked with goat serum, and dewaxed with xylene and gradient ethanol. The sections were then treated at 4 °C for an overnight period with primary antibodies to ACSL4 (1:200, Proteintech, USA), Collagen II (1:200, Novus, USA), Aggrecan (1:500, Servicebio, China), and Piezo1 (1:200, Affinity, USA). The sections were exposed to goat anti-rabbit IgG-HRP secondary antibody for 1 h at room temperature the next day. The DAB Substrate kit (ZLI-9018, ZSGB) was used for detection, and the samples were then counterstained with 1% hematoxylin. Using a microscope (Leica DMI3000B, Germany), pictures were recorded. ImageJ quantified the areas that were positive.

### Statistical analysis

Statistical analyses were performed using GraphPad Prism 9.4.0 (GraphPad Software, USA). Two-tailed unpaired *t*-tests were used to analyze the two groups. Multiple comparisons were analyzed using the one-way analysis of variance. All results are expressed as the mean ± SEM. *P* < 0.05 was considered statistically significant. *n* ≥ 3 for all samples. Each experiment was repeated independently three times.

### Supplementary information

Supplemental Materials

## Data Availability

All data are available from the corresponding authors upon reasonable request.
