# Supplementary material for: Piezo1 channel exaggerates ferroptosis of nucleus pulposus cells by mediating mechanical stress-induced iron influx
Source: Bone Res. 2024 Mar 29;12:20. doi: 10.1038/s41413-024-00317-9 (PMC10980708; doi:10.1038/s41413-024-00317-9)
Supplement: Supplementary file 1 — Supplemental Materials [file 41413_2024_317_MOESM1_ESM.docx]

**Supplementary Information**

Piezo1 channel exaggerates ferroptosis of nucleus pulposus cells by mediating mechanical stress-induced iron influx

Ziqian Xiang^1,2,#^, Pengfei Zhang^1,#^, Chunwang Jia^1,#^, Rongkun Xu^1^, Dingren Cao^6^, Zhaoning Xu^7^, Tingting Lu^8^, Jingwei Liu^9^, Xiaoxiong Wang^1,2^, Cheng Qiu^1^, Wenyang Fu^1^, Weiwei Li^5^, Lei Cheng^1,^, Qiang Yang^4^, Shiqing Feng^1,3^, Lianlei Wang^1,*^, Yunpeng Zhao^1,*^, Xinyu Liu^1,*^

This supporting information includes:

Materials and methods

Supplementary Figures S1 to S9

Supplementary Table S1 to S3

**Materials and methods**

**Analysis of RNA-sequencing data**

Total RNA was extracted using RNAprep Pure Plant Plus Kit (DP441, Tiangen) and purified by RNAClean XP Kit (A63987, Beckman Coulter) and RNase-Free DNase Set (79254, Qiagen). Libraries were constructed using U-mRNAseq Library Prep Kit (AT4221, Kaitai-Bio) with Ribo-off rRNA Depletion Kit (N407, Vazyme). All RNAs were quantified as FPKM (Fragments Per Kilobase Million Mapped Reads) by StringTie.  The formula was defined as FPKM = 106×F/ (NL×10-3), where F is the number of fragments assigned to a certain gene in a certain sample, N is the total number of mapped reads in the certain sample, and L is the length of the certain gene.

Differentially expressed genes were determined by edgeR, and genes with FDR <0.05 and |log2 foldchange| >1 were identified as differentially expressed genes. GO and KEGG enrichment analysis were performed for all differentially expressed mRNAs. GO enrichment analysis showed the biological functions of differentially expressed mRNAs. KEGG (Kyoto Encyclopedia of Genes and Genomes, [http://www.kegg.jp/](http://www.kegg.jp/)) is a database resource that contains a collection of manually drawn pathway maps representing our knowledge of the molecular interaction and reaction networks. Using the same method as GO enrichment analysis, significantly enriched KEGG pathways were identified.

**CCK8**

Cell viability was examined using Cell Counting Kit-8 (C0038, Beyotime, China). In 96-well microplates (Corning, USA), cells were grown at a density of 5 × 10^3^/ well in 100 L of media before being exposed to mechanical or chemical stimuli for a set number of hours (0, 6, 12, 24, 36, 48, and 72). Then 10 μL of CCK8 reagent was added to each well and cultured for 2 h. A microplate reader (Bio-Rad, USA) was used to analyze the absorbance at 450 nm. The survival percentage relative to control group cells was expressed by the absorbance.

**Actin-tracker**

The cytoskeleton was labeled by Actin-Tracker kit (C2201S, Beyotime, China). Briefly, cells were incubated with phalloidin (1:1000) according to the instructions and cell nuclei were stained with 4′, 6-diamidino-2-phenylindole (DAPI; C1005, Beyotime, China). The fluorescence information was acquired by fluorescence microscope (Olympus, Japan).

**Calcein-AM/PI staining**

The dual staining of live and dead cells was performed using the Calcein-AM/PI double stain kit (C2015M, Beyotime, China) in line with the manufacturers instructions. NPCs were stimulated by different stimulations. After incubation for 24 h, NPCs were treated with a dyeing working solution containing 2 M calcein-AM and 4.5 M PI for 15 min at room temperature and in the dark. They were the examined under an inverted fluorescence microscope (Olympus, Japan).

**
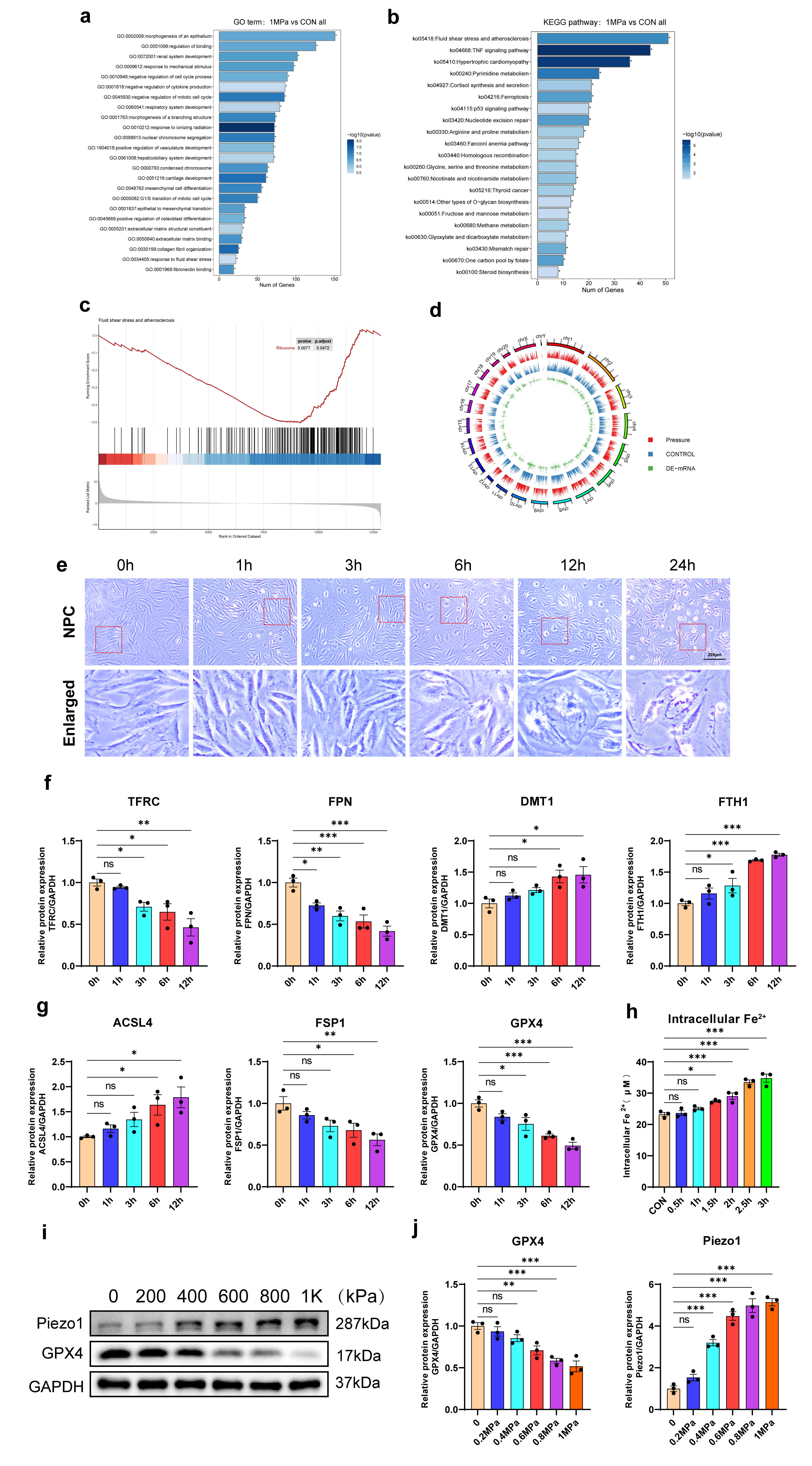
Supplementary Figure S1.**

**Supplementary Fig. S1 Mechanical stress changed the markers of iron metabolism and ferroptosis in a time-dependent manner**. **a-d** RNA sequencing analysis in rat NPCs treated with 1 MPa mechanical stress in a Ca^2+^-free medium. n = 3 replicates. **a** GO enrichment analysis of NPCs. **b** KEGG enrichment analysis of differentiated pathways in NPCs. **c** GSEA analysis of sequencing data of NPCs. **d** Circle map analysis of NPCs. **e** Representative morphological changes of NPCs were shown at 0, 1, 3 ,6 12, and 24 h after 1 MPa mechanical stimulation. Scale bar, 200 μm. **f,g** Protein expressions of NPCs at different time-points after 1 MPa mechanical stimulation in a Ca^2+^-free medium were quantified using Image J software. **h** Intracellular Fe^2+^ content are measured by Iron Assay Kit at different time-points. n = 3 replicates. **i,j** Western blot analysis of Piezo1 and GPX4 in different mechanical stress conditions and quantification. GAPDH was used as an internal control. n = 3 replicates. All data are expressed as the mean ± SEM, n=3 replicates from one representative of 3 independent experiments. ns (no significance), *P < 0.05, **P < 0.01, ***P < 0.001.

**Supplementary Figure S2.**

**
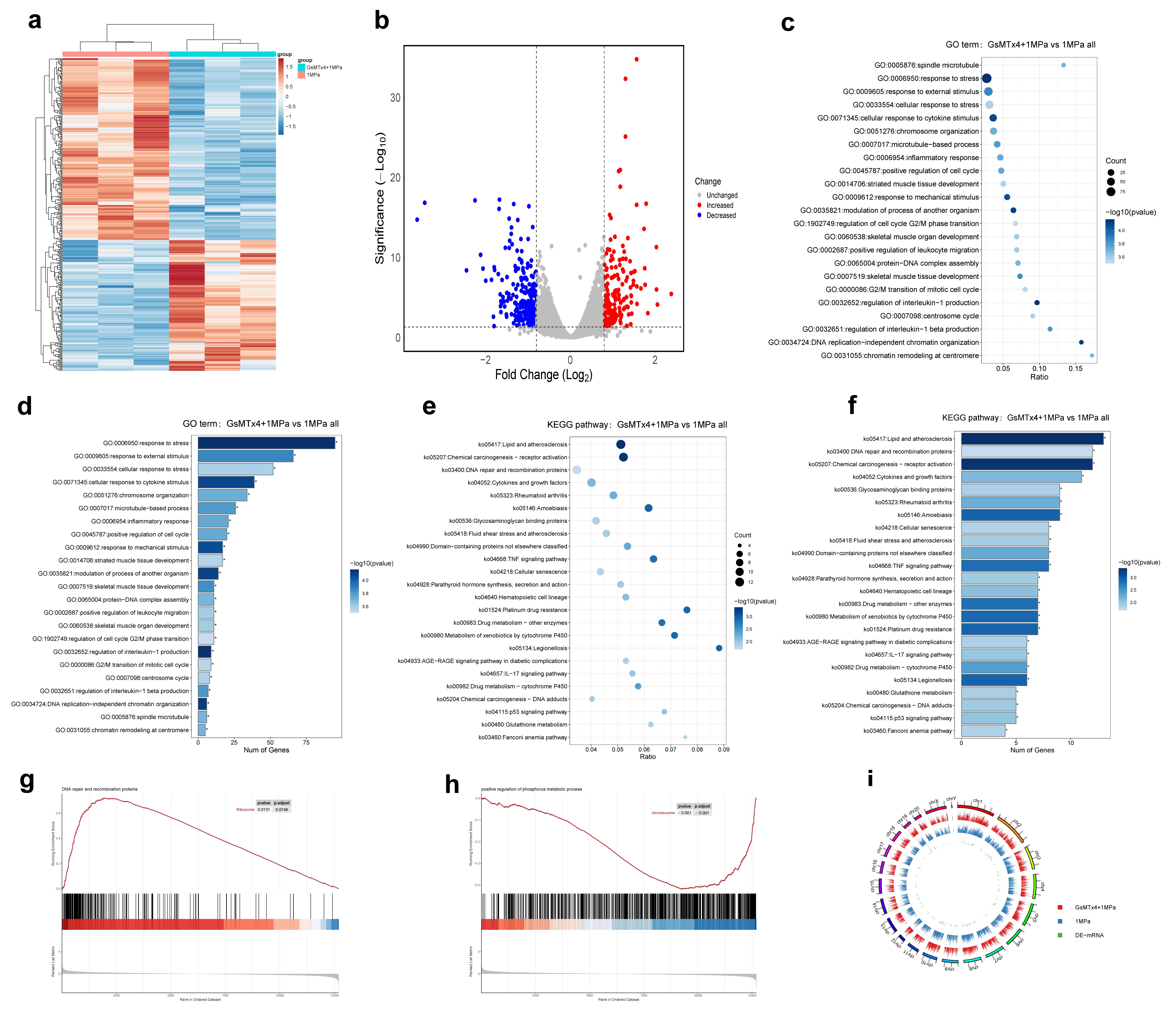
**

**Supplementary Fig. S2 Piezo1 inhibition alleviated the mechanical stress-induced damage.** **a-i** RNA sequencing analysis in rat NPCs from the GsMTx4 +1 MPa groups and those from the 1 MPa stress groups. n = 3 replicates. **a** Heatmap illustrating the different genes expression in NPCs. **b** Volcano plot show the differentially expressed genes of the two groups. **c-d** GO analysis in NPCs. **e-f** KEGG enrichment analysis of differentiated pathways. **g**,**h** GSEA analysis showing the changes in NPCs. **i** Circle map analysis of NPCs.

**Supplementary Figure S3.**

**
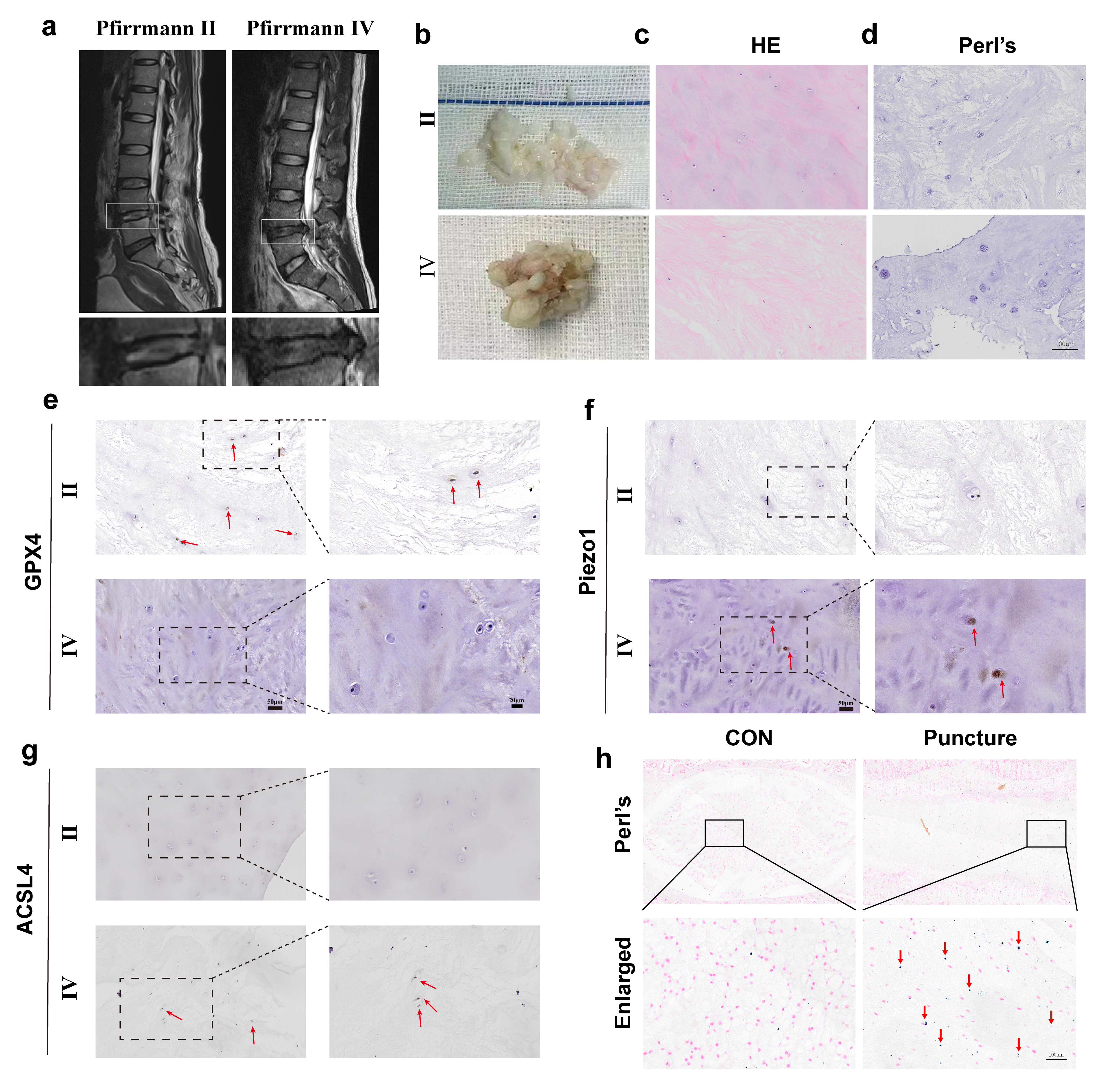
**

**Supplementary Fig. S3 Iron overload was involved in IVD degeneration**. **a** Representative human MR T2-weighted images of grade II (left panel) and grade IV (right panel) IVDs. **b** General view of NP tissues from donors accepted surgery. **c**, **d** The HE and Perl’s staining of NP tissues from human degenerated discs. Scale bar, 100 μm. **e-g** The IHC staining of GPX4, Piezo1 and ACSL4 in human NP sections. Scale bar, 50 μm. **h** The Perl’s prussian blue staining of NP tissues from a needle puncture IVDD model and normal control rats. Arrows indicate Perl’s-positive cells. Scale bar, 100 μm.

**Supplementary Figure S4.**

**
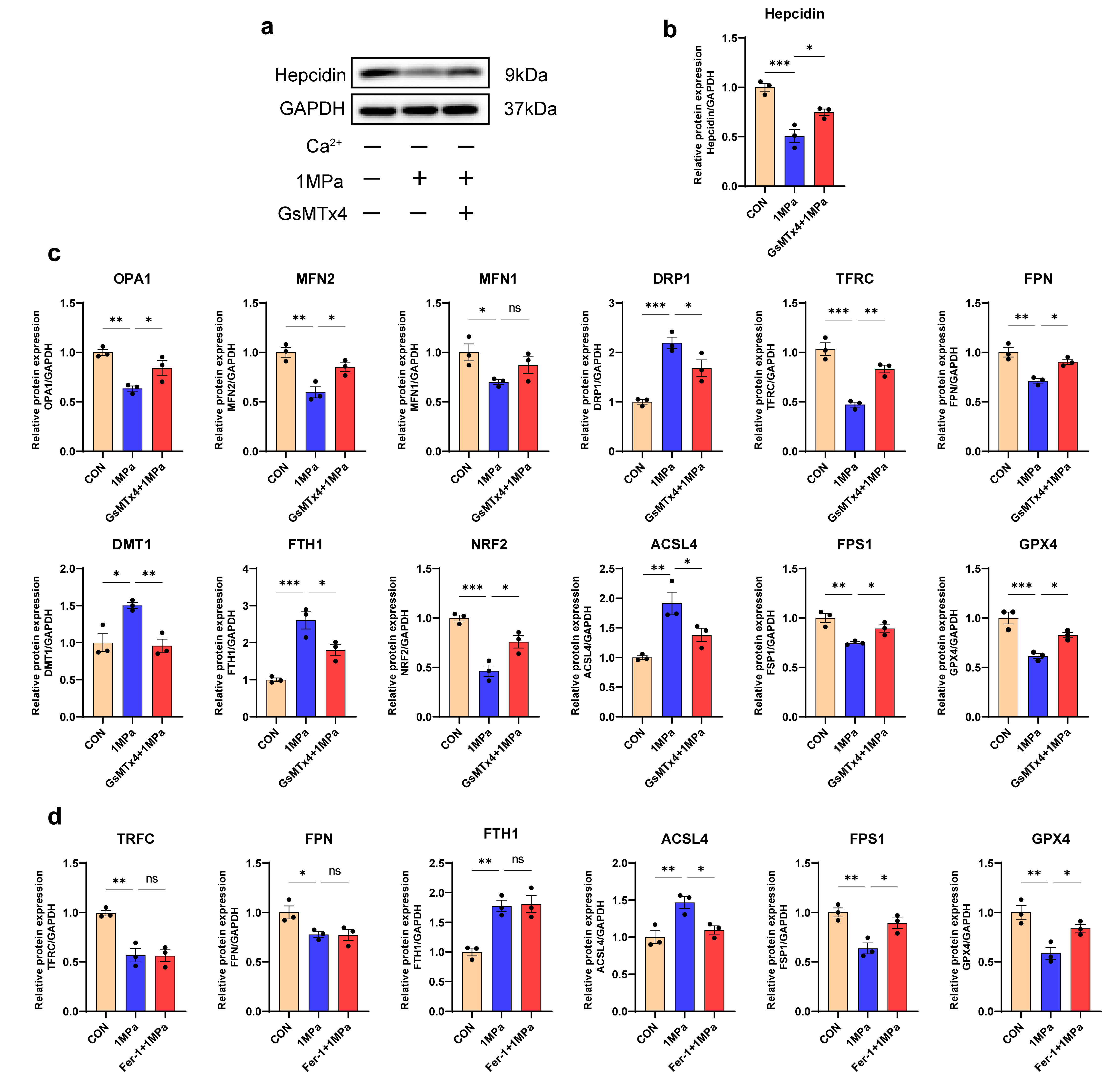
**

**Supplementary Fig. S4 Protein expressions after GsMTx4 and Fer-1 treatment under 1 MPa mechanical stress**. **a** Protein expressions of NPCs treated with or without GsMTx4 under 1 MPa mechanical stress for 24 h were quantified using ImageJ software. n = 3 replicates. **b** Protein expressions of NPCs treated with or without Fer-1 under 1 MPa mechanical stress for 24 h were quantified using ImageJ software. All data are expressed as the mean ± SEM, n=3 replicates from one representative of 3 independent experiments. ns (no significance), *P < 0.05, **P < 0.01, ***P < 0.001.

**Supplementary Figure S5.**

**
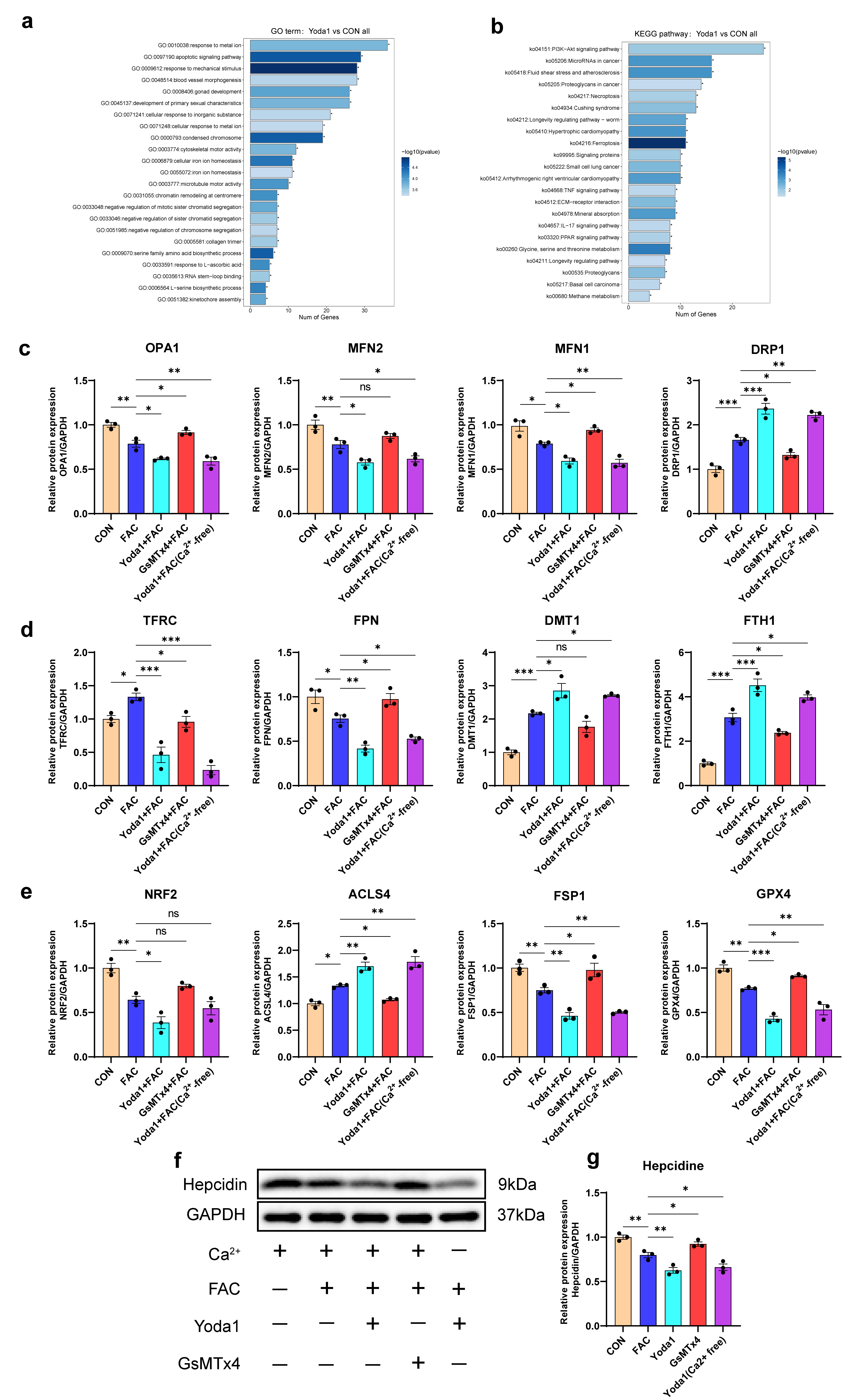
**

**Supplementary Fig. S5 Piezo1 activation plays an important role in the occurrence of ferroptosis. a**,**b** GO and KEGG enrichment analysis in NPCs treated with Yoda1 in a Ca^2+^-free medium. **c-g** Protein expressions of NPCs treated with Yoda1 or GsMTx4 in 100 μM FAC for 24h were quantified using ImageJ software. All data are expressed as the mean ± SEM, n=3 replicates from one representative of 3 independent experiments. ns (no significance), *P < 0.05, **P < 0.01, ***P < 0.001.

**Supplementary Figure S6.**


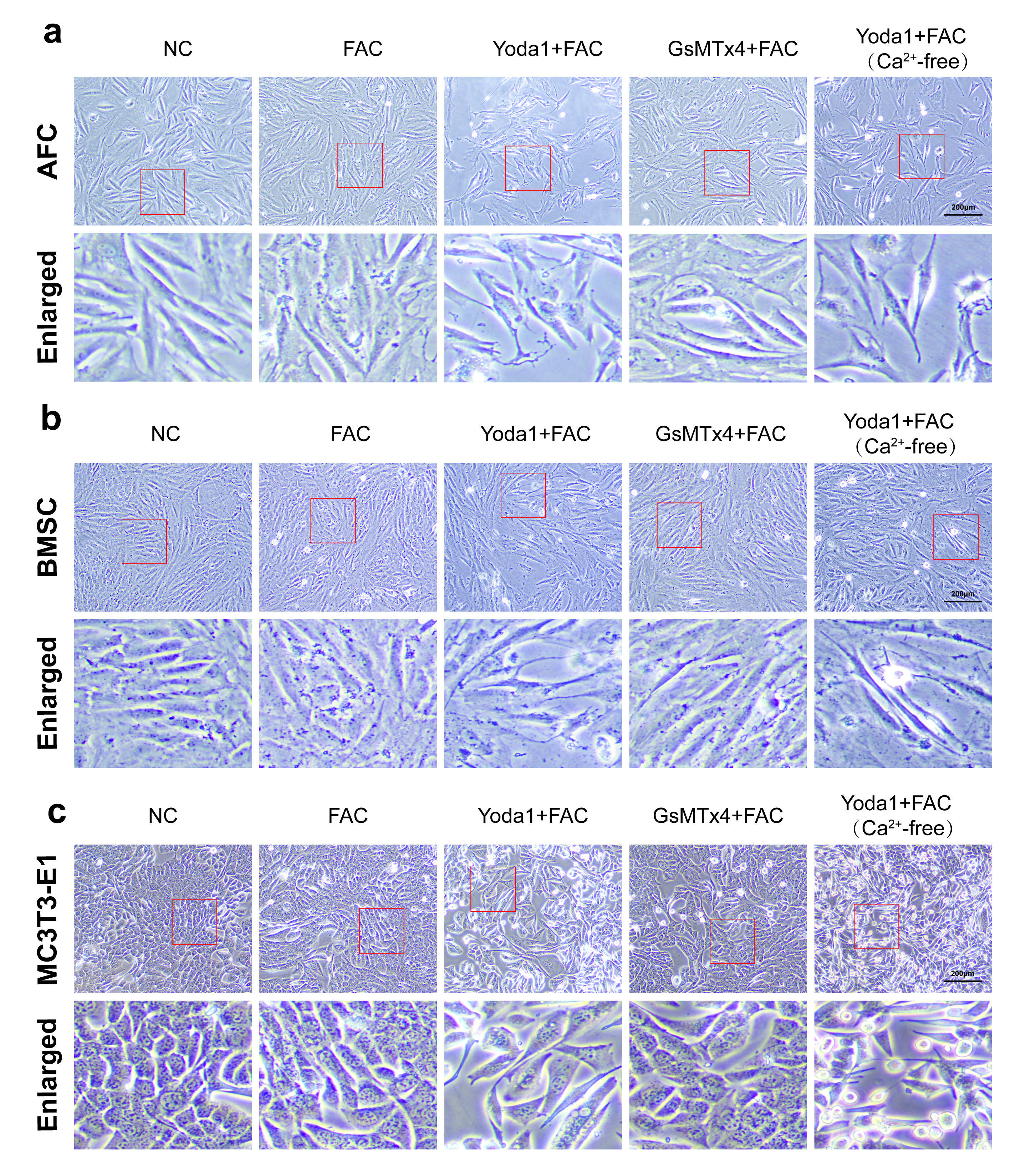


**[Figure S6.](https://www.nature.com/articles/s42255-022-00602-z/figures/15)** **The effect of Piezol activation or inhibition on other types of cells.** **a-c** Representative morphological changes of AFCs, BMSCs and MC3T3-E1 were shown treated with or without the stimulation of Yoda1 and GsMTx4 under high iron environment for 24 h. Scale bar, 200 μm..

**Supplementary Figure S7.**


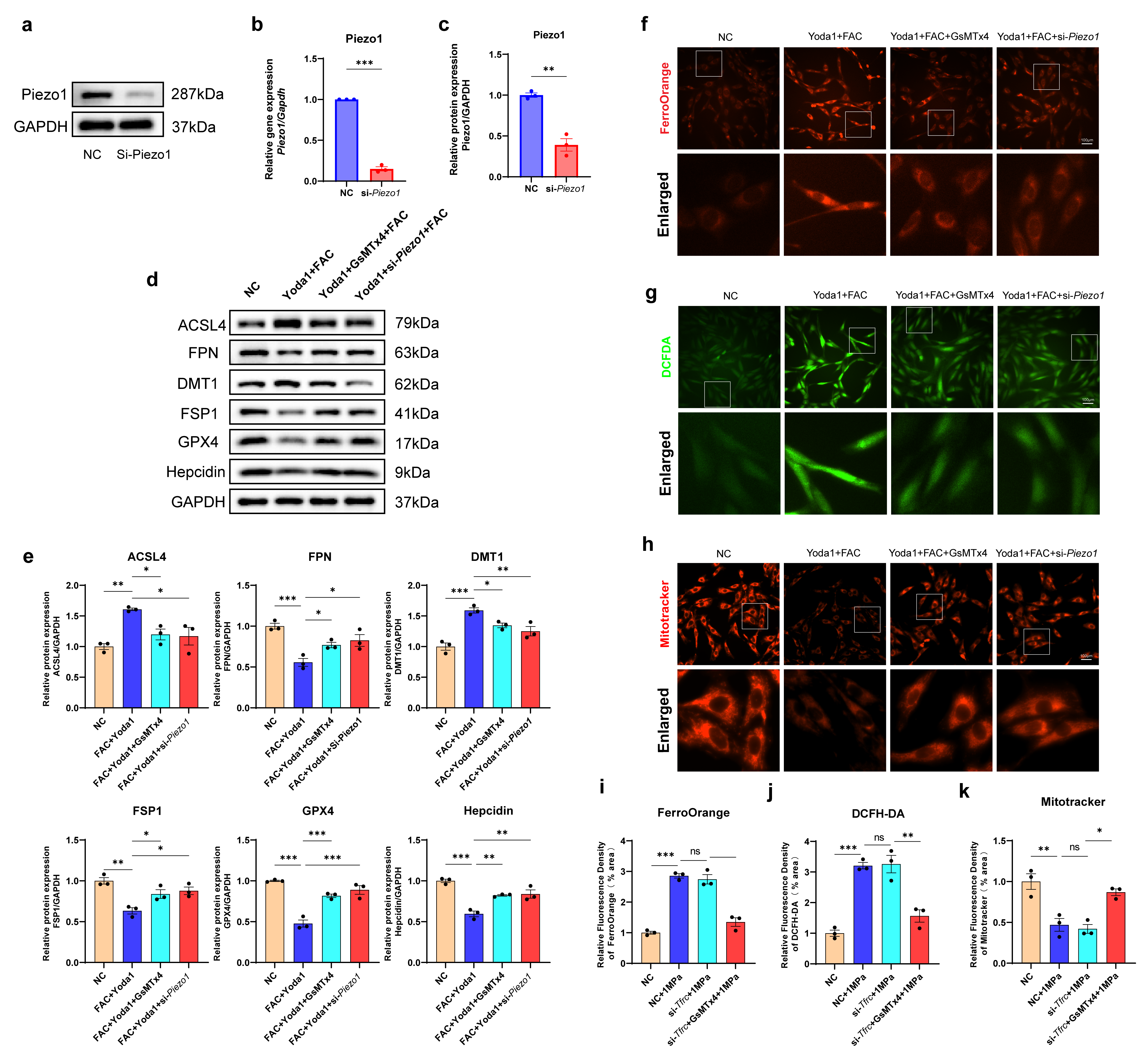


**Supplementary Fig. S7 Specific blockade of piezo1 reversed the effects of Yoda1 in NPCs**.

**a-c** PCR and WB analysis of NPCs after transfection of si-*Piezo1* for 24 hours and quantitation. n = 3 replicates. **d,e** WB analysis of different markers of NPCs aftre treated with Yoda1+FAC in the presence of GsMTx4 or si-*Piezo1 for* for 24 h and quantitation. GAPDH was used as an internal control. n = 3 replicates. **f-k** Representative images of NPCs were taken using FerroOrange, DCFH-DA and Mitotracker after different treatment for 24 h. **T**he relative MFI was analysed by ImageJ software. n = 3 replicates. Scale bar, 100 μm. All data are expressed as the mean ± SEM, n=3 replicates from one representative of 3 independent experiments. ns (no significance), *P < 0.05, **P < 0.01, ***P < 0.001.

**Supplementary Figure S8.**

**
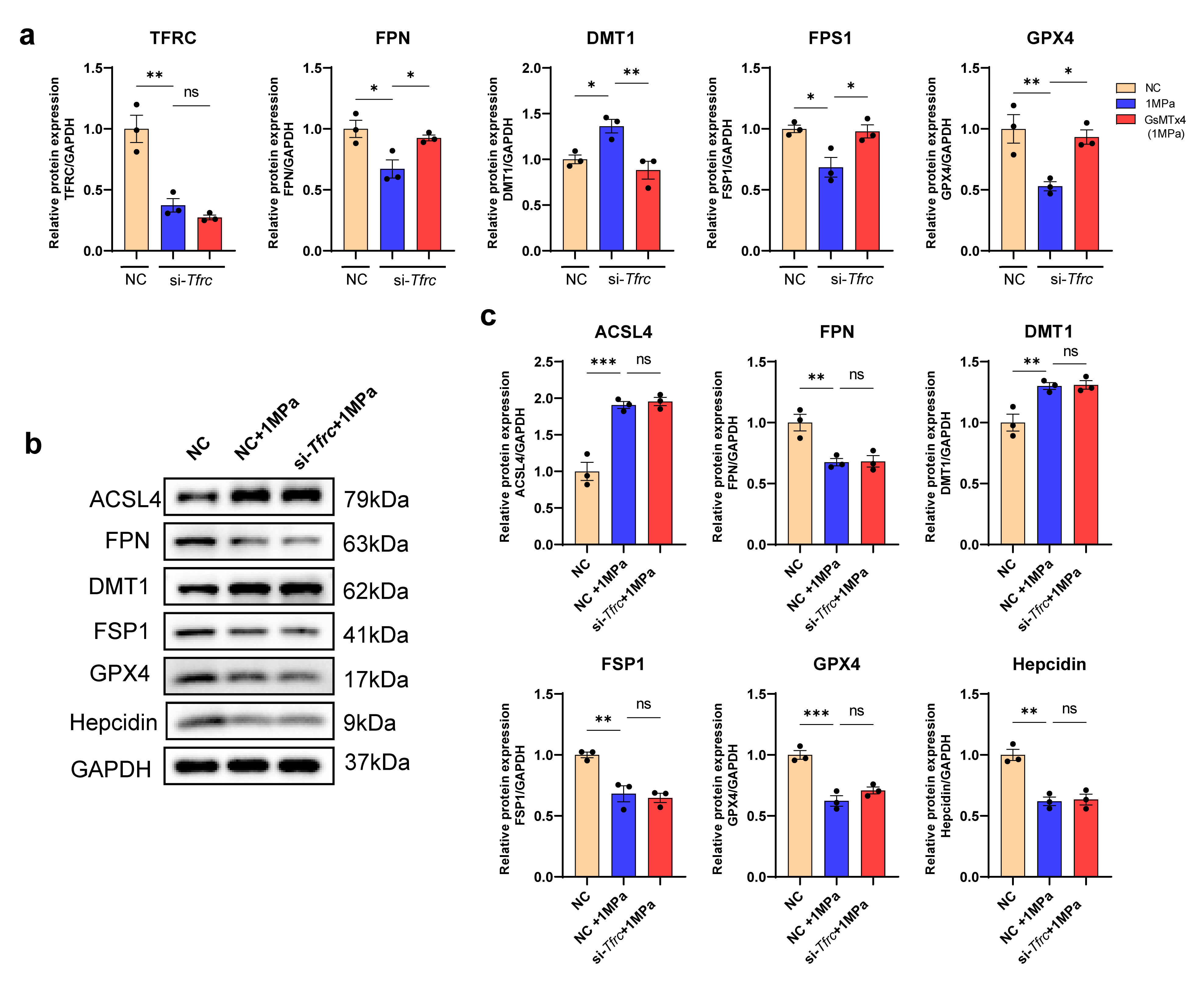
**

**Supplementary Fig. S8 Protein expressions after *Tfrc* KD**. Protein expressions of NPCs treated with or without GsMTx4 in with 1 MPa mechanical stress for 24 h after transfection of si-*Tfrc* were quantified using ImageJ software. **b,c** Western blot analysis of NPCs after transfection of NC or si-*Tfrc* under 1 MPa mechanical stress and quantification. GAPDH was used as an internal control. n = 3 replicates. All data are expressed as the mean ± SEM, n=3 replicates from one representative of 3 independent experiments. ns (no significance), *P < 0.05, **P < 0.01, ***P < 0.001.

**Supplementary Figure S9.**

**
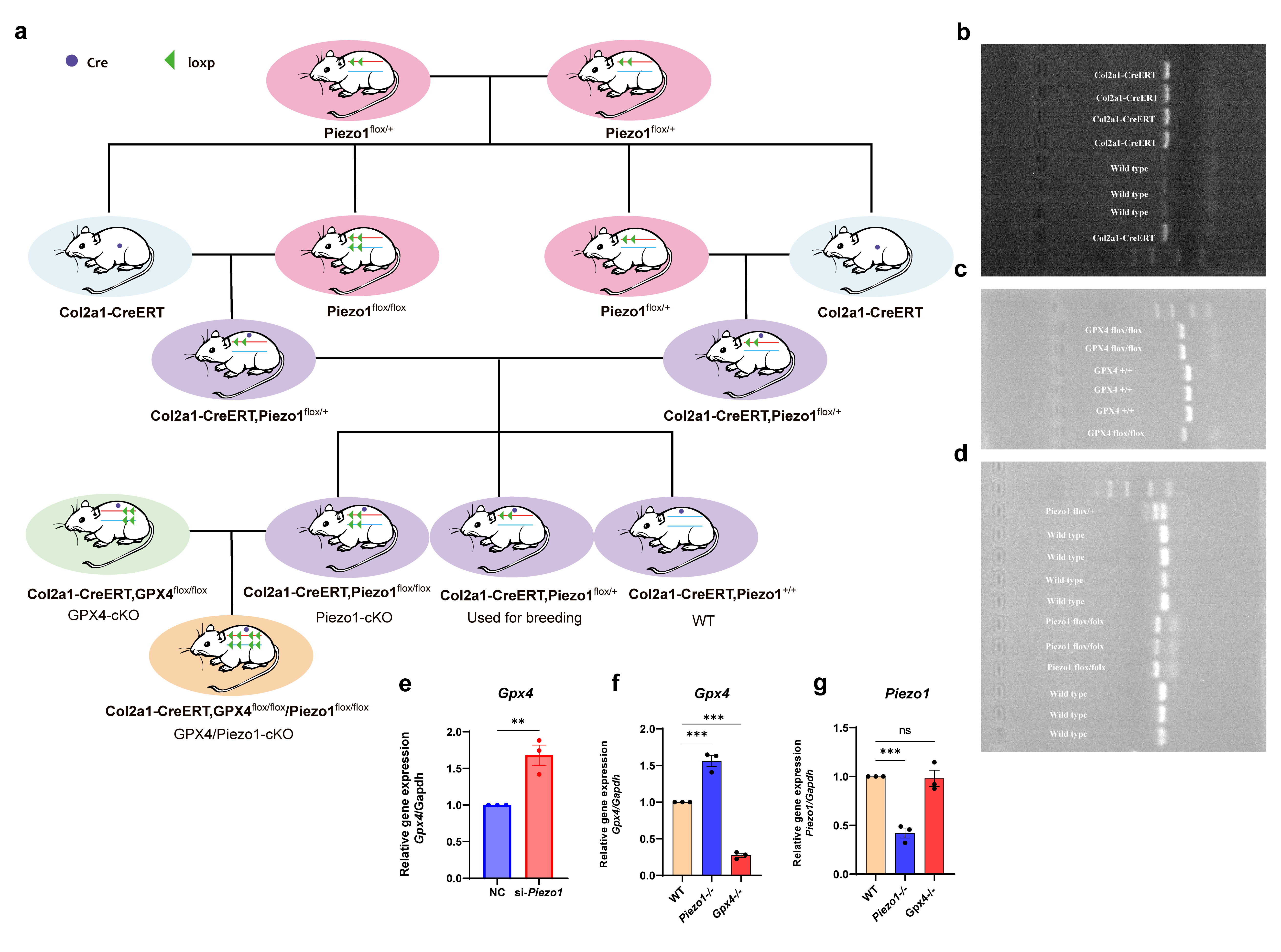
**

**Supplementary Fig. S9 Breeding strategies and gene identification of mice**. **a** Breeding strategies for GPX4-cKO, Piezo1flox/flox: Col2-CreERT (Piezo1-cKO) and GPX4/Piezo1-cKO mice. **b-d** Gene identification results: Homozygotes (Gpx4^flox/flox^) :238bp. Heterozygotes (Gpx4^flox/+^) 238/204bp. Wild type (Gpx4^+/+^) 204bp; Homozygotes (Piezo1^flox/flox^) :210bp. Heterozygotes (Piezo1^flox/+^) 210/142bp. Wild type (Piezo1^+/+^) 142bp; Col2a1- CreERT: 358bp. Wild type: No stripe. **e** PCR analysis of *Gpx4* of NPCs after transfection of si-*Piezo1* for 24 h and quantification. *Gapdh* was used as an internal control. n = 3 replicates. **f,g** PCR analysis of *Gpx4* and *Piezo1* of WT, *Piezo1*-cKO and *Gpx4*-cKO mice. *Gapdh* was used as an internal control. n = 3 replicates. All data are expressed as the mean ± SEM, n=3 replicates from one representative of 3 independent experiments. ns (no significance), *P < 0.05, **P < 0.01, ***P < 0.001.

**Supplementary Table S1. Primers used for si-*Tfrc* and si-*Piezo1***

| **Target** | **Forward Primers,5’-3’** | **Reverse Primers,5’-3’** |
| --- | --- | --- |
| **Tfrc-Rat-1914**  **Piezo1-Rat-753** | GCCGGUCAGUUCAUUAUUATT  GGAGGAGGAUGACAUAGAUTT | UAAUAAUGAACUGACCGGCTT  AUCUAUGUCAUCCUCCUCCTT |

**Supplementary Table S2. Primers used for qPCR**

| **Target** | **Forward Primers,5’-3’** | **Reverse Primers,5’-3’** |
| --- | --- | --- |
| ***Tfrc***  ***Fpn***  ***Dmt1***  ***Fth1***  ***Nrf2***  ***Acsl4***  ***Fsp1***  ***Gpx4***  ***Gapdh*** | TACGTTCCCCGTTGTTGAGG  CCCCTGCTCTGGCTGTAAAA  TGGCAGTGTTTGATTGCGTT  AACTACCACCAGGACTCGGA  ATTCCCAGCCACGTTGAGAG  TGTGAGCGCATACCTGGATT  GGGAGAAGGACAGACGAAGC  CTCGCAATGAGGCAAAACCG  TCTCTGCTCCTCCCTGTTCT | CCAGATGACTGAGATGGCGG  TCGAAAAGTGCGGAAGGGTT  CCGCTGGTATCTTCGCTCA  CTTCAGGGCCACATCATCCC  TCCTGCCAAACTTGCTCCAT  CAGCCGTAGGTAAAGCAGGA  TCTTCCGGGGCTCCTTATCT  GGGAAGGCCAGGATTCGTAA  ATCCGTTCACACCGACCTTC |

**Supplementary Table S3. Primers used for Gene identification**

| **Target** | **Forward Primers,5’-3’** | **Reverse Primers,5’-3’** |
| --- | --- | --- |
| ***Gpx4^flox/flox^***  ***Piezo1^flox/flox^***  ***Col2a1-CreERT*** | TCCATTGGTCGGCTGCGTGAGG  AGCAAGGCCAATGTAGTATCTGG  CACTGCGGGCTCTACTTCAT | ACCCTGGATACGGTGACCCGAC  CTATTGGTGCCTAGTTGGCAGAC  ACCAGCAGCACTTTTGGAAG |
